# Extensive transcriptional and chromatin changes underlie astrocyte maturation in vivo and in culture

**DOI:** 10.1038/s41467-021-24624-5

**Published:** 2021-07-15

**Authors:** Michael Lattke, Robert Goldstone, James K. Ellis, Stefan Boeing, Jerónimo Jurado-Arjona, Nicolás Marichal, James I. MacRae, Benedikt Berninger, Francois Guillemot

**Affiliations:** 1 Neural Stem Cell Biology Laboratory, The Francis Crick Institute, London, UK; 2 Advanced Sequencing Facility, The Francis Crick Institute, London, UK; 3 Metabolomics Science Technology Platform, The Francis Crick Institute, London, UK; 4 Software Development & Machine Learning Team, The Francis Crick Institute, London, UK; 5 Bioinformatics & Biostatistics, The Francis Crick Institute, London, UK; 6 Institute of Psychiatry, Psychology & Neuroscience, Centre for Developmental Neurobiology, King’s College London, London, UK; 7 MRC Centre for Neurodevelopmental Disorders, King’s College London, London, UK; 8 Institute of Physiological Chemistry, University Medical Center of the Johannes Gutenberg University Mainz, Mainz, Germany; 9 The Francis Crick Institute, London, UK

**Keywords:** Developmental biology, Molecular biology, Neuroscience, Cellular neuroscience, Development of the nervous system

## Abstract

Astrocytes have essential functions in brain homeostasis that are established late in differentiation, but the mechanisms underlying the functional maturation of astrocytes are not well understood. Here we identify extensive transcriptional changes that occur during murine astrocyte maturation in vivo that are accompanied by chromatin remodelling at enhancer elements. Investigating astrocyte maturation in a cell culture model revealed that in vitro-differentiated astrocytes lack expression of many mature astrocyte-specific genes, including genes for the transcription factors Rorb, Dbx2, Lhx2 and Fezf2. Forced expression of these factors in vitro induces distinct sets of mature astrocyte-specific transcripts. Culturing astrocytes in a three-dimensional matrix containing FGF2 induces expression of *Rorb*, *Dbx2* and *Lhx2* and improves astrocyte maturity based on transcriptional and chromatin profiles. Therefore, extrinsic signals orchestrate the expression of multiple intrinsic regulators, which in turn induce in a modular manner the transcriptional and chromatin changes underlying astrocyte maturation.

## Abstract (web summary)

Astrocytes have functions crucial for brain homeostasis, which are disrupted in many neurological disorders, but how these functions are established during astrocyte maturation is largely unknown. Here the authors show transcriptional and chromatin changes underlying astrocyte maturation in mice and identify transcription factors regulating maturation of cultured astrocytes.

## Introduction

Astrocytes are the most abundant glial cells in the mammalian central nervous system and they serve essential functions in brain development and homoeostasis. Astrocytes are generated from neural stem cells (NSCs) during late embryonic and early postnatal stages. First, a ‘gliogenic switch’ enables NSCs to progress from neurogenesis to the generation of astrocytes and oligodendrocytes. Specified glial precursors then migrate from the progenitor zones to the brain parenchyma where they proliferate and differentiate during early postnatal stages^1,2^. During a subsequent phase of maturation in the first few postnatal weeks, immature astrocytes exit the cell cycle and aquire a fully mature phenotype^1^.

The molecular mechanisms controlling astrocyte specification and the early steps of differentiation of immature astrocytes have been intensely investigated and diverse regulators of these processes have been identified^3–6^. In contrast, little is known of the mechanisms controlling the later step of maturation of immature postnatal astrocytes into fully mature adult astrocytes.

Postnatal maturation is associated with major changes in astrocyte biology. Before their maturation, astrocytes contribute to brain vascularisation, establishment of the blood-brain-barrier, axon pathfinding and establishment and elimination of synapses^7^. Maturing astrocytes then acquire new functions required for adult brain homoeostasis. They provide metabolic and trophic support for neurons, regulate the blood-brain-barrier and local cerebral blood flow, and modulate neuronal activity^7,8^.

Although the functions of astrocytes change profoundly during their maturation, the regulatory mechanisms underlying these changes have only begun to be examined. This analysis has been performed mostly in in vitro models, including cultures of acutely isolated postnatal astrocytes, cultures of NSCs differentiated with agonists of the BMP or JAK-STAT signalling pathways^9–12^, and pluripotent stem cell-derived three dimensional neural cultures or spheroids^13^. Several mechanisms have been shown to promote maturation-associated transcriptional changes in these cell culture models, including the factors Runx2 and p53, the FGF signalling pathway and cell–cell-contacts^11,14,15^. However, in vitro-differentiated astrocytes are thought to remain immature^15–17^, and it is, therefore, unclear whether the mechanisms identified so far are sufficient to account for the complete maturation of astrocytes in vivo.

To gain insights into the mechanisms driving astrocyte maturation, we have characterised acutely isolated murine astrocytes at both postnatal and adult stages, as well as in vitro differentiated astrocytes. We show that astrocyte maturation is promoted by extrinsic signals that induce multiple transcription factors that act largely independently to regulate distinct gene expression modules that together promote a mature astrocytic phenotype. This comprehensive characterisation of gene expression and chromatin accessibility changes in vivo and in vitro constitutes a valuable resource for future research, which we make accessible on an interactive online platform (https://biologic.crick.ac.uk/astrocyte).

## Results

### Astrocyte maturation in vivo proceeds through transitional stages

**Fig. 1:**
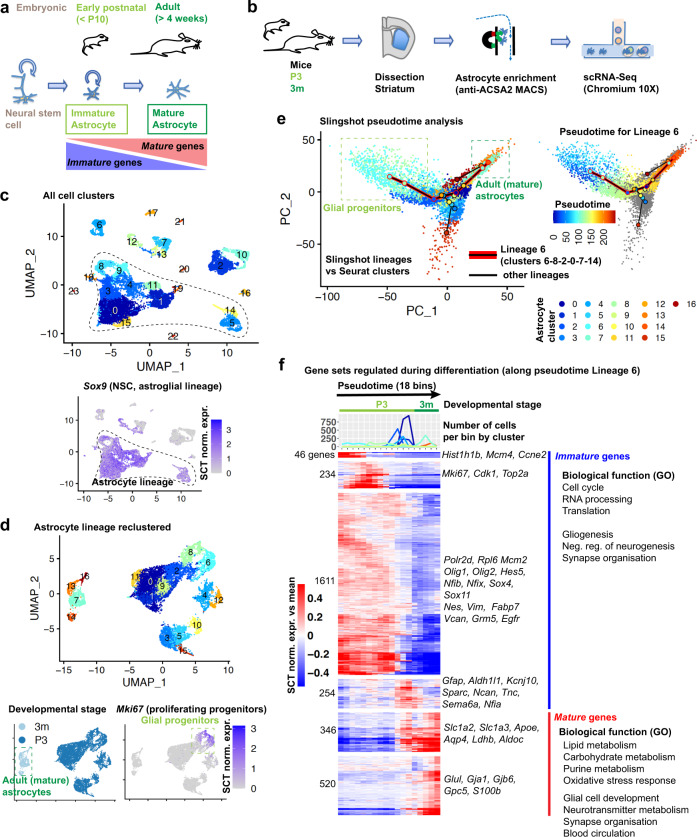
Astrocyte maturation in vivo proceeds through intermediate stages. **a** Current model of astrocyte differentiation in mice: neural stem cells generate immature astrocytes that proliferate until around postnatal day 10 (P10) and become fully mature astrocytes by 4 weeks of age. **b** Approach for single-cell RNA-Seq analysis of striatal astrocyte maturation: immature astrocytes from the striatum of postnatal day 3 (P3) mice and mature astrocytes from the striatum of 3-months-old (3m) mice are enriched by magnetic bead assisted cell sorting (MACS) for the astrocyte-specific ACSA2 antigen, followed by single-cell RNA sequencing (scRNA-Seq). **c** Uniform Manifold Approximation and Projection (UMAP) dimension reduction plots of combined single-cell transcriptomes from postnatal and adult striatum. Expression of *Sox9* is projected on the UMAP plot in the lower graph. **d** UMAP plot of *Sox9*+ cells highlighted in (**c**) and reclustered. The lower graphs show cells’ origin by developmental stage and expression of *MKi67* projected on UMAP plot. **e** Pseudotime analyis of clusters in (**d**) shown projected on a principle component analysis (PCA) plot. Left: cluster assignment and the lineage relationships identified by Slingshot (starting with the proliferating progenitor cluster 6). Right: pseudotime progression of cells included in the pseudotime trajectory connecting the progenitor cluster 6 with putative mature adult astrocyte clusters 7 and 14 (most different/distant along principle component 1 (PC1)), via the early postnatal clusters 8, 2 and 0 (Lineage 6). **f** Temporally-expressed genes along the pseudotime trajectory shown in (**e**). The heatmaps show the mean relative expression of each gene in all cells in each pseudotime bin (number of cells from each cluster shown above). Genes were ordered by hierarchical clustering and grouped into gene sets with similar expression patterns. Example genes and potential biological functions based on gene ontology (GO) analysis are shown. 3011 temporally expressed genes were detected with tradeSeq by comparing the 5894 cells assigned to the main pseudotime trajectory (Lineage 6) from (**e**). Gene expression was normalized using the Seurat SCTtransform algorithm (SCT norm. expr.). See also Supplementary Fig. 1, Supplementary Data 1.

To gain a better understanding of the molecular mechanisms underlying astrocyte maturation, we examined astrocytes isolated from mice at an early postnatal stage (postnatal day 3; P3), when astrocytes have been specified but remain immature and partially proliferating, and at a young adult stage (~3 months), when astrocytes have reached full maturity (Fig. 1a). We collected tissues from the early postnatal and adult striatum, which we dissociated and enriched for astrocytes using magnetic-activated cell sorting (MACS) with the astrocyte surface antigen ACSA2^18^, and performed single-cell RNA-Sequencing (scRNA-Seq) using the 10X Genomics Chromium platform (Fig. 1b). We sequenced a total of 16,690 cells from three independent preparations each of early postnatal and adult tissues, of which 14,373 were retained after filtering for low quality cells (Supplementary Data 1). To identify cell types, we performed dimension reduction with the Uniform Manifold Approximation and Projection (UMAP) approach implemented in Seurat 3. This analysis revealed 24 cell clusters that we defined using a panel of established lineage markers, with only minor batch effects (Fig. 1c, Supplementary Fig. 1a, b). 9992 astroglial lineage cells, identified by expression of *Sox9*^19^ and other well-established early and late astrocyte markers, separated into several clusters (Fig. 1c, Supplementary Fig. 1a–c), which we isolated and re-clustered (Fig. 1d). This led to the identification of two putative proliferating progenitor clusters expressing the proliferation marker *Mki67* (clusters 6 and 8 of the re-clustered astroglial subset), 11 clusters of *Mki67*-negative early postnatal astrocytes (0 to 5, 9 to 12 and 15) and four clusters of adult astrocytes (7, 13, 14 and 16).

To infer putative lineage relationships, we performed pseudotime analysis of these cells using the Slingshot algorithm (Fig. 1e, f, Supplementary Data 1). Using the proliferating progenitor cluster 6 as a starting point, this analysis revealed one trajectory (Lineage 6) terminating in the main adult clusters 7 and 14, via the second *Mki67*-positive cluster 8 and the two early postnatal *Mki67*-negative clusters 2 and 0. From this trajectory, other astrocyte clusters branched off at several points. To further characterise these clusters, we compared them with a reference scRNA-Seq atlas containing juvenile (P20-P30) astrocytes from various brain regions^20^ (Supplementary Fig. 1d).

The cells of the P3 astrocyte clusters 1, 9 and 11 were very similar to the main lineage cluster 0 (Fig. 1d, e) and were interspersed among the main telencephalic astrocyte clusters from the reference dataset (Supplementary Fig. 1d). In contrast, the cells of two main side branches in the Slingshot analysis (P3 clusters 3, 4, 5, 10, 12, 15 and adult clusters 13 and 16) clustered with non-telencephalic populations of the reference dataset (Supplementary Fig. 1d), suggesting that they may represent contaminating astrocytes from adjacent brain regions.

To identify gene expression changes during striatal astrocyte maturation, we focussed on cells in Lineage 6 (Fig. 1e, f). Using the tradeSeq algorithm, we identified 3011 dynamically expressed genes, which we grouped based on hierarchical clustering of their pseudotemporal expression into 6 gene sets with distinct expression patterns (Fig. 1f, Supplementary Data 1). Four of these gene sets showed highest expression during early or intermediate pseudotime stages and contained 2145 *immature striatal astrocyte genes*, while two gene sets showed highest expression at the end of the pseudotime trajectory and contained 866 *mature striatal astrocyte genes* (Fig. 1f, Supplementary Data 1).

We next investigated the potential biological functions of these immature and mature astrocyte genes, using the literature and Gene Ontology analysis (Fig. 1f, Supplementary Data 1). The immature astrocyte gene sets included predominantly genes of the basal transcription and translation machineries and genes involved in cell proliferation, e.g. *Mcm2*, *Mki67* and *Cdk1*. Immature astrocyte genes were also enriched for GO terms linked to neuronal and glial development, such as “cerebral cortex development”, “negative regulation of neurogenesis“, “positive regulation of gliogenesis” and “axon regeneration”, and included transcription factors involved in astrocyte differentiation (*Nfia, Nfib* and *Hes5*) and oligodendrocyte differentiation (*Olig1, Olig2*). In contrast, the mature astrocyte genes were enriched for GO terms linked to lipid and carbohydrate metabolism, as well as terms associated with adult astrocyte functions, such as ‘synapse organization’, ‘regulation of neurotransmitter levels’ and ‘blood circulation’ and included many genes known to be involved in such functions, such as *Glul* (glutamine synthetase), *Gjb6* (Cx30), *Aldoc, Apoe*, *Aqp4* and the glial glutamate transporters *Slc1a3* (Glast) and *Slc1a2* (GLT-1) (Fig. 1f, Supplementary Data 1).

Overall, these transcriptional changes support the hypothetical astrocyte differentiation trajectory shown in Fig. 1e, f and suggest that proliferating and potentially bipotent progenitors differentiate to fully mature astrocytes through at least two intermediate stages that are postmitotic but still immature. The gene expression analysis suggests that during differentiation and maturation, astrocytes first downregulate programmes involved in proliferation and neuronal and glial development, before inducing genes involved in adult astrocyte functions.

### Comparison of striatal and cortical datasets reveals a common astrocyte maturation signature

**Fig. 2:**
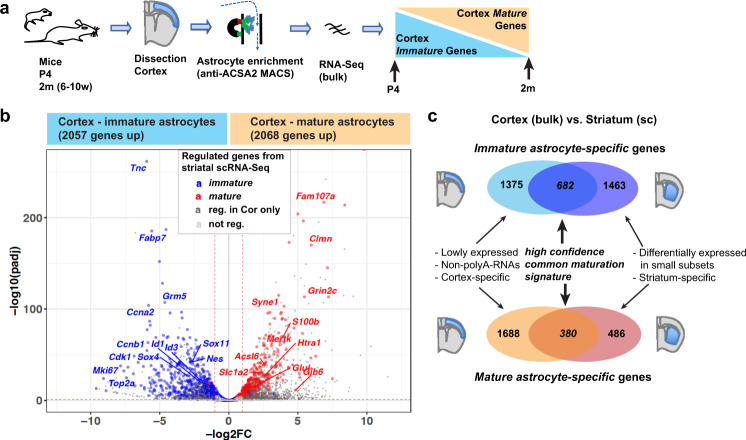
Comparison of striatal and cortical datasets reveals a common astrocyte maturation signature. **a** Approach used for MACS (magnetic bead assisted cell sorting) enrichment and bulk RNA-Sequencing (RNA-Seq) analysis of immature astrocytes from postnatal day 4(P4) cortex and of mature astrocytes from adult cortex (age 2 months (2m), range 6–10 weeks (6–10w)). **b**, **c** Volcano plot (**b**) and Venn diagrams (**c**) show genes differentially expressed between cortical astrocyte preparations from P4 and adult brain. Maturation-regulated genes identified by scRNA-Seq of striatal astrocytes (see Fig. 1f) are highlighted in (**b**). Venn diagrams (**c**) show overlap of genes significantly regulated between early postnatal and adult astrocyte preparations in both striatal scRNA-Seq and cortical bulk RNA-Seq datasets. Biological and technical differences between the analyses may contribute to the differences in genes detected in the two datasets, as indicated in (**c**). Differential genes: DESeq2 analysis, each *n* = 3; two-sided Wald test with Benjamini–Hochberg correction; significance threshold: adjusted *p*-value (padj) ≤ 0.05, absolute log2(fold change)(log2FC) ≥ 1.See also Supplementary Data 2.

We next performed a bulk RNA-Seq analysis of astrocytes isolated from the grey matter of the cerebral cortex at P4, when astrocytes are still immature, and at 2 months of age, when they are mature (Fig. 2a). We performed this complementary analysis to increase the sensitivity of our transcriptional analysis of astrocyte maturation, and to determine which maturation-regulated genes identified by the scRNA-Seq approach are part of a generic astrocyte maturation programme (common to striatal and cortical astrocytes).

Using DESeq2, we detected 2057 genes that were downregulated and 2068 genes that were upregulated in cortical astrocytes between P4 and 2 months (Fig. 2b, c, Supplementary Data 2). Of the immature striatal astrocyte genes detected by scRNA-Seq analysis, 32% were significantly downregulated in cortical astrocytes between P4 and 2 months. Reciprocally, 44% of the mature striatal astrocyte genes were significantly upregulated in adult cortical astrocytes. Most other immature and mature striatal astrocyte genes showed a similar trend of regulation during maturation of cortical astrocytes, although not reaching statistical thresholds (Fig. 2b, c, Supplementary Data 2).

The large differences between cortical and striatal maturation-regulated gene sets likely reflect mainly differences in the approaches used to obtain them, and only to a lesser extent genuine biological differences between the tissues (Fig. 2c). Indeed, unifying the analysis by merging the striatal single-cell transcriptomes into pseudo-bulk RNA-Seq datasets, revealed a much larger overlap of genes differentially regulated in the two tissues (Supplementary Fig. 2a). 71% of the genes enriched in early postnatal cortical astrocytes were also enriched in early postnatal striatal astrocytes, and 65% of the genes enriched in adult cortical astrocytes were also enriched in adult striatal astrocytes. However, the genes enriched in the adult bulk RNA-Seq data also contain many oligodendrocyte genes, because adult, but not early postnatal astrocyte preparations, contain a significant proportion of oligodendrocytes (Supplementary Fig. 1a–c). To eliminate this contamination, we focussed our further analyses on astrocyte maturation-regulated genes validated both by single cell analysis of striatal astrocytes (thus removing the contaminating oligodendrocytes) and in the cortical bulk RNA-Seq analysis. These included 380 genes that were upregulated from early postnatal astrocytes to adult astrocytes in the two brain regions (Fig. 2b, c, Supplementary Data 2), thus representing a high confidence set of *mature astrocyte-specific genes* (from hereon also referred to as *mature genes* for brevity). Similarly, 682 genes were consistently downregulated in adult astrocytes and represent high confidence *immature astrocyte-specific genes* (from hereon also called *immature genes*). Using cortical astrocyte preparations for further bulk analyses was appropriate as the oligodendrocyte contamination appears much lower in cortical than striatal preparations (Supplementary Fig. 2b).

To assess the extent to which the maturation programme identified in the cerebral cortex and striatum is recapitulated in other brain regions, we further compared the striatal scRNA-Seq data with the reference dataset^20^ (Supplementary Figs. 1d, 2c). Hierarchical clustering showed that the expression of *immature* and *mature astrocyte-specific* signature genes is overall similar in adult striatal astrocytes and in juvenile telencephalic and non-telencephalic astrocytes from the reference dataset (Supplementary Fig. 2c), suggesting that large parts of the astrocyte maturation gene expression programme identified by scRNA-Seq in the striatum also operate during maturation of astrocytes in other brain regions.

Finally, we validated the temporal expression pattern of some *immature* and *mature* genes by immunolabelling together with the pan-astrocytic marker Sox9 at P4 and 2 months of age (Supplementary Fig. 3). In line with their RNA expression profiles (Supplementary Fig. 3a), the proliferation marker Ki67 (*Mki67*) is expressed in a subset of immature astrocytes, while Id1, Id3, BLBP (*Fabp7*) and Nes are broadly expressed in the striatum and cortex at P4, and all these *immature* markers are absent at 2 months of age (Supplementary Fig. 3b–d). The *mature* markers GLT-1 (*Slc1a2*) and GS (*Glul*) show limited expression already at P4 (Supplementary Fig. 3d,e), while Cx30 (*Gjb6*) and S100b are only detectable at 2 months. Overall this analysis at protein level recapitulates the maturation-associated changes observed at the RNA level.

### Maturation-associated chromatin remodelling at putative regulatory elements

**Fig. 3:**
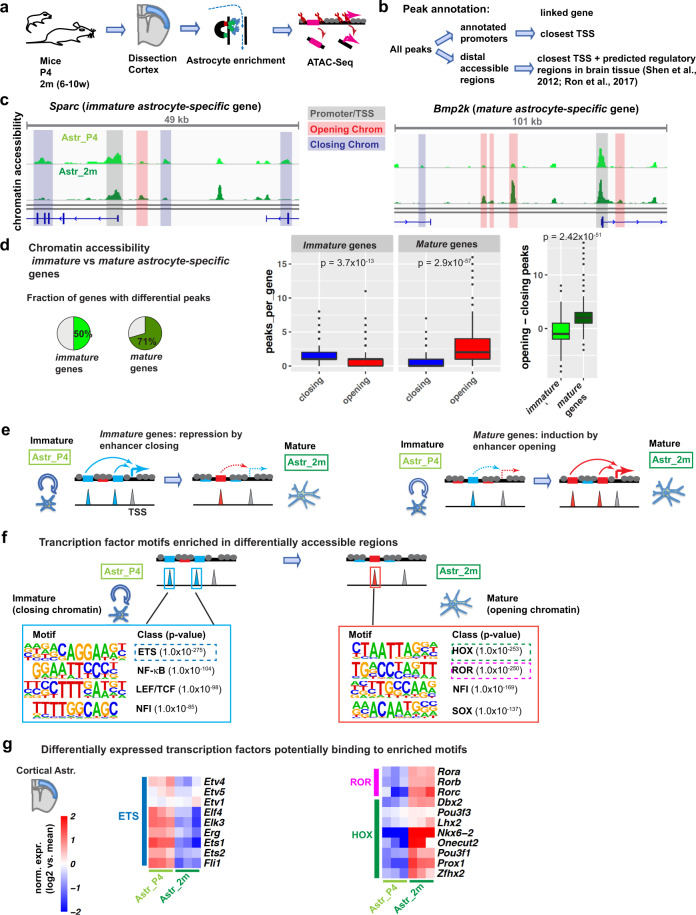
Maturation-associated chromatin remodelling at putative regulatory elements. **a** Approach used for MACS enrichment (magnetic bead assisted cell sorting) and ATAC-Seq (assay for transposase-accessible chromatin using sequencing) analysis of immature and mature astrocytes from P4 and 2-month-old cortices, respectively (Astr_P4, Astr_2m), in order to identify stage-specific accessible chromatin regions, i.e. putative regulatory elements. **b** Strategy for mapping ATAC-Seq peaks to putative target genes. **c** Genome tracks show chromatin accessibility around the transcription start sites (TSS) of the *immature astrocyte-specific* gene *Sparc* and of the *mature astrocyte-specific* gene *Bmp2k*. Reduced size of ATAC-Seq peaks in adult vs P4 astrocytes indicates closing chromatin, while increased peak size indicates opening chromatin. **d**, **e** Genes regulated during astrocyte maturation (from Fig. 2c) are globally associated with chromatin regions that change in their accessibility between P4 and adult stages (**d**). This suggests a model whereby changes in enhancer accessibility contribute to changes in gene expression during astrocyte maturation (**e**). **f** Transcription factor binding motifs enriched in closing and opening chromatin, identified by de novo motif enrichment analysis. The most enriched motifs are similar to ETS, homeodomain (HOX) and retinoic acid receptor-related orphan receptor (ROR) transcription factor binding motifs. **g** Expression in cortical astrocytes, based on bulk RNA-Seq analysis in Fig. 2, of selected transcription factors that bind to motifs highlighted in (**f**). Displayed are all ETS factors enriched in immature astrocytes, and all ROR and HOX factors enriched in mature astrocytes, either in the cortical (bulk) or the striatal (single cell) RNA-Seq dataset. Expression of the factors regulated along pseudotime in striatal astrocytes is shown in Supplementary Fig. 5c. Statistical analysis and data presentation: **c** Tracks represent merged reads of 3 or 4 replicates (for P4 and 2 m respectively); **c**, **d** Differential peaks: DESeq2 analysis, *n* = 3 (Astr_P4), *n* = 4 (Astr_2m); two-sided Wald test with Benjamini–Hochberg correction; significance threshold: adjusted *p*-value ≤ 0.05, absolute log2(fold change) ≥ 1; **d** Two-sided Wilcoxon Rank Sum test (*n* = 341 immature vs 268 mature genes with associated differential peaks). Boxplots show: centre line, median; box limits, upper and lower quartiles; whiskers, 1.5× interquartile range; points, outliers. **g** Differential genes: DESeq2 analysis, each *n* = 3; two-sided Wald test with Benjamini–Hochberg correction; significance threshold: adjusted *p*-value ≤ 0.05, absolute log2(fold change) ≥1; Shown are log2-transformed mean centred, normalized expression values. See also Supplementary Figs. 4 and 5, Supplementary Data 3–5.

To gain insights into the molecular control of astrocyte maturation, we next investigated the mechanisms that may regulate the differential expression of the *immature* and *mature astrocyte-specific* genes. Chromatin remodelling establishes stable transcriptional changes during cellular differentiation processes. In particular, chromatin opening is required for non-pioneer transcription factors to bind and activate cell-type-specific enhancers^21^. We investigated the changes in chromatin accessibility taking place during astrocyte maturation by performing assay for transposase-accessible chromatin using sequencing (ATAC-Seq)^22^ in cortical astrocytes isolated at P4 and 2 months (Fig. 3a). A total of 95,066 peaks were called on the merged ATAC-Seq dataset. Removal of small peaks that were not clearly distinguishable from the background retained 56,219 highly reproducible peaks (see ‘Methods’, Supplementary Fig. 4a and Supplementary Data 3). 56% of the astrocyte ATAC peaks overlapped with peaks from a reference analysis of brain tissue by DNase-Seq (Encode database, Supplementary Fig. 4b, c), an alternative method to detect open chromatin, further demonstrating the high quality of the data. Many ATAC peaks covered annotated transcriptional start sites (TSS) (called below ‘promoters’) but most were located in intergenic regions and introns (Supplementary Data 4). 44% of TSS-distal ATAC peaks overlapped with the enhancer-enriched histone mark H3K4me1 in reference brain ChIP-Seq datasets (Encode database; Supplementary Fig. 4b,c), suggesting that a large fraction of distal ATAC peaks are located in enhancer elements. To identify genes that might be regulated by these candidate enhancers, we used a closest TSS-based approach (Fig. 3b, Supplementary Fig. 4d, e). We also integrated published regulatory regions from mouse brain tissue^23,24^, which identified additional potential enhancers located at great distance from the genes they regulate (Fig. 3b, Supplementary Fig. 4d, e; Supplementary Data 4). Using the ATAC-Seq datasets, annotations in the Ensembl database and reference datasets, we compiled a list of 15,216 accessible promoters, 22,501 accessible enhancers and 18,502 additional distal accessible regions in immature and/or mature astrocytes (Supplementary Fig. 4f, Supplementary Data 4).

Comparing the ATAC-Seq datasets from immature and mature astrocytes revealed prominent changes in chromatin accessibility between these stages, particularly in distal accessible regions (Fig. 3c, Supplementary Fig. 4a, d, f). Differentially accessible regions were associated with 50% of *immature astrocyte-specific* genes, such as *Sparc*, and 71% of *mature* genes, such as *Bmp2k* (Fig. 3c, d, Supplementary Fig. 4a, Supplementary Data 5). *Immature* genes were predominantly associated with regions more accessible at P4 than at 2 months (closing chromatin) while *mature* genes were predominantly associated with elements more accessible at 2 months (opening chromatin; Fig. 3c, d, Supplementary Data 5). These results suggest that changes in enhancer accessibility may contribute to the transcriptional changes taking place during astrocyte maturation (Fig. 3e). Similar results were obtained with a more stringent selection of candidate enhancers that considered only open chromatin regions up to a distance of 100 kb from the nearest TSS (Supplementary Fig. 5a, Supplementary Data 4), consistent with most promoter-enhancer-interactions occurring within this distance^25^.

To assess more directly the extent to which accessibility changes at promoters, as compared to enhancers, may regulate gene expression during astrocyte maturation, we assessed promoters and enhancers of immature and mature astrocyte genes separately (Supplementary Fig. 5b). Only a small fraction of regulated genes (less than 15%) showed changes in promoter accessibility, and promoter opening occurred almost exclusively at genes induced during maturation, and promoter closing occurred at downregulated genes. Changes at enhancers concerned many more regulated genes, and also correlated with changes in gene expression, although accessibility at some distal elements was anti-correlated to gene expression, suggesting a repressive function or incorrect annotation.

We next aimed to identify transcription factors that may drive changes in gene expression underlying astrocyte maturation. For this, we performed motif enrichment analysis in differentially accessible regions using the Homer software. In chromatin regions that closed between P4 and 2 months, the most enriched binding motifs included those for ETS, NF-κB and LEF/TCF transcription factors (Fig. 3f). In chromatin regions that opened between postnatal and adult stages, motifs associated with homeodomain (HOX) and retinoic acid receptor-related orphan receptor (ROR) factors were most enriched (Fig. 3f). Motifs for the key astrogliogenic NFI transcription factors were found enriched in both opening and closing chromatin (Fig. 3f).

Examining the expression of transcription factors associated with the enriched motifs in the cortical bulk RNA-Seq dataset (Fig. 3g) and the striatal sc-RNA-Seq dataset (Supplementary Fig. 5c), revealed that, consistent with the changes in binding site enrichment, several ETS genes had a higher expression in early postnatal than adult astrocytes. Reciprocally, *Rora*, *Rorb*, and several HOX genes including *Dbx2*, *Lhx2* and *Pou3f3* (*Brn1*) were upregulated in adult astrocytes (Fig. 3g, Supplementary Fig. 5c). Altogether, our data suggest that transcription factors from the ETS, HOX and ROR families may direct chromatin remodelling events and thereby contribute to the transcriptional changes associated with astrocyte maturation.

### In vitro differentiation of NSCs into astrocytes fails to recapitulate in vivo maturation

**Fig. 4:**
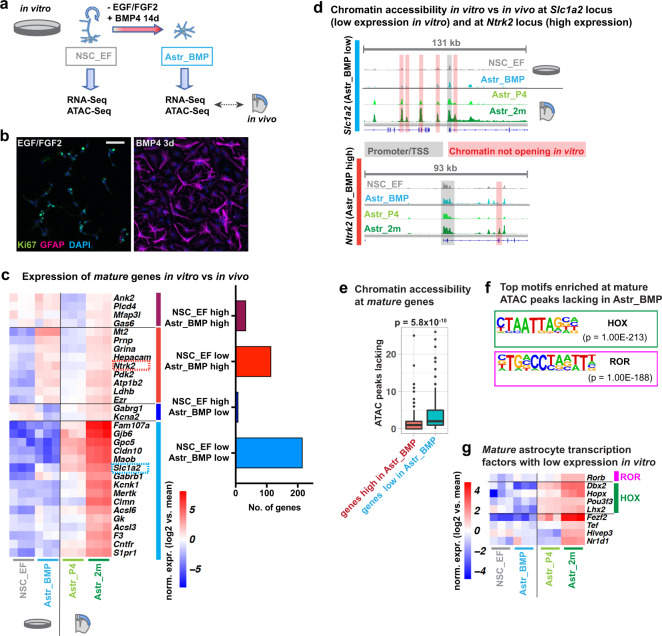
In vitro differentiation of NSCs into astrocytes fails to recapitulate in vivo maturation. **a** NSCs maintained in EGF/FGF2-containing medium (NSC_EF) are differentiated into astrocytes by culture in BMP4-containing medium for 14 days (Astr_BMP); maturity of astrocytes in these cultures is assessed by RNA-Seq and ATAC-Seq analysis and comparison with datasets from P4 and adult cortical astrocytes (Astr_P4/Astr_2m from Figs. 2 and 3). **b** Immunolabeling for the proliferation marker Ki67 and the astrocyte marker GFAP after 3 days of differentiation; scale bar 50 μm. Representative images from 3 biological replicates. **c** Expression of *mature astrocyte-specific* genes (from Fig. 2c) in cultured astrocytes and brain astrocytes. Heatmap of selected genes and number of genes, grouped by their expression patterns in cultured astrocytes. ‘NSC_EF/Astr_BMP low’ refers to significantly lower expression compared to Astr_2m, ‘high’ to equal or higher expression. **d** ATAC-Seq genome tracks show chromatin accessibility in cultured astrocytes vs brain astrocytes around the transcriptional start site (TSS) of *Slc1a2* and *Ntrk2* (expression highlighted in (**c**)). **e** *Mature astrocyte-specific* genes with low expression in vitro (Astr_BMP) lack accessibility at sites open in vivo (Astr_2m), more than genes with high expression. **f** Transcription factor binding motifs enriched in DNA regions where ATAC-Seq analysis shows lower chromatin accessibility in cultured astrocytes compared to adult brain astrocytes (de novo motif enrichment analysis). **g** Expression of selected transcription factors induced during astrocyte maturation in vivo and expressed at low levels in cultured astrocytes. Highlighted are factors that might bind the motifs in (**f**). Heatmap of cortical astrocyte bulk RNA-Seq data from Fig. 2; the factors displayed are differentially expressed in cortical and striatal astrocytes (except *Lhx2* and *Pou3f3* only in striatal astrocytes). Expression of the factors in striatal astrocytes is shown in Supplementary Fig. 6e. Statistical analysis and data presentation: **c**, **g** Differential genes/peaks: DESeq2 analysis; each *n* = 3 (*n* = 4 for ATAC-Seq Astr_2m data); two-sided Wald test with Benjamini–Hochberg correction; significance threshold: adjusted *p*-value ≤ 0.05, absolute log2(fold change) ≥ 1; Heatmaps show log2-transformed mean centred, normalized expression values; **d** Tracks represent merged reads of 3 replicates (4 replicates for Astr_2m). **e** Two-sided Wilcoxon Rank Sum test (*n* = 211 with low vs 143 genes with high expression in vitro). Boxplots show: centre line, median; box limits, upper and lower quartiles; whiskers, 1.5× interquartile range; points, outliers. See also Supplementary Fig. 6, Supplementary Data 6.

To examine the activity of candidate transcription factors in promoting astrocyte maturation, we turned to an in vitro model of astrocyte differentiation. We established NSC cultures from the early postnatal cortex, a likely source of cortical astrocytes in vivo^2^, and from the adult subventricular zone, expanded them in the presence of EGF and FGF2, and subsequently differentiated them into astrocytes by growth factor withdrawal and addition of BMP4^9,10^ (Fig. 4a).

After 3 days in differentiation medium, NSCs had downregulated the proliferation marker Ki67 and begun to express the astroglial lineage marker GFAP (Fig. 4b). Cortex- and adult subventricular zone-derived NSCs were differentiated into astrocytes in the presence of BMP4 for 14 days to allow time for maturation, and were then analysed by RNA-Seq. Their transcriptional profile was compared with that of undifferentiated NSCs in culture and with the RNA-Seq datasets obtained from cortical astrocytes acutely isolated from mice at P4 and 2 months (Fig. 2).

As expected, many astrocyte markers were upregulated in in vitro-differentiated astrocytes compared to NSCs, including *Aldh1l1*, *Sox9*, *Gfap*, and *Aqp4*, which reached expression levels comparable to those observed in cortical astrocytes in 2-months old brains (Supplementary Fig. 6a). To compare the maturation state of in vitro-differentiated astrocytes with that of adult brain astrocytes, we next analysed the expression of *immature* and *mature astrocyte-specific* genes (Fig. 2). Astrocytes differentiated in vitro expressed 40% of *mature* genes at levels similar to those observed at 2 months in vivo, but 60% were expressed at lower levels or not at all, including key effector genes of astrocyte functions such as *Gjb6*(Cx30) and *Slc1a2*(GLT-1) (Fig. 4c, Supplementary Data 6).

Compared to the limited induction of *mature* genes, the differentiation protocol was more efficient at repressing *immature* genes. In vitro-differentiated astrocytes expressed 72% of *immature* genes at low levels, similar to those found in mature astrocytes in vivo and only 28% were expressed at high levels as in immature astrocytes in vivo (Supplementary Fig. 6b). Comparison of our dataset for in vitro-differentiated astrocytes with published data for cultured primary astrocytes^17^ and astrocytes differentiated with CNTF^11^ showed that astrocytes in different in vitro models present similarly low expression levels of many *mature astrocyte-specific* genes (Supplementary Fig. 6c). Overall these results suggest that in vitro-differentiated astrocytes do not reach a level of maturation comparable to that of adult brain astrocytes.

We next performed ATAC-Seq to investigate changes in chromatin accessibility associated with in vitro differentiation and compare accessible sites with those observed in adult brain astrocytes. At the *Slc1a2* locus, a *mature* gene expressed only at low levels in vitro (Fig. 4c, d), many sites which had large ATAC peaks in adult brain astrocytes presented only small signals in cultured astrocytes (Fig. 4d, Supplementary Fig. 6d). In contrast, the *Ntrk2* locus, which is expressed highly in both in vitro-differentiated and adult brain astrocytes, showed more similar accessibility profiles in the in vitro and brain samples (Fig. 4c, d, Supplementary Fig. 6d). Globally, *mature* genes that were expressed at low levels in in vitro-differentiated astrocytes were lacking more accessibility peaks than genes highly expressed in cultured cells (Fig. 4e). Motif enrichment analysis revealed that the sequences most enriched in regions with reduced accessibility in cultured astrocytes were HOX- and ROR-binding motifs (Fig. 4f), which were also enriched in chromatin regions opening between P4 and 2m in brain astrocytes (Fig. 3f). Together our results show that in vitro-differentiated astrocytes fail to gain chromatin accessibility at many regulatory elements associated with *mature astrocyte-specific* genes. The results also suggest that a lack of activity of ROR and HOX transcription factors in in vitro-differentiated astrocytes might prevent chromatin opening at key regulatory regions and may block full maturation. Supporting this hypothesis, we found that several ROR and HOX genes induced during astrocyte maturation in vivo remained expressed at low levels in in vitro-differentiated astrocytes, including *Rorb*, *Dbx2* and *Lhx2* (Fig. 4g, Supplementary Fig. 6e).

### Different transcription factors control distinct aspects of astrocyte maturation

**Fig. 5:**
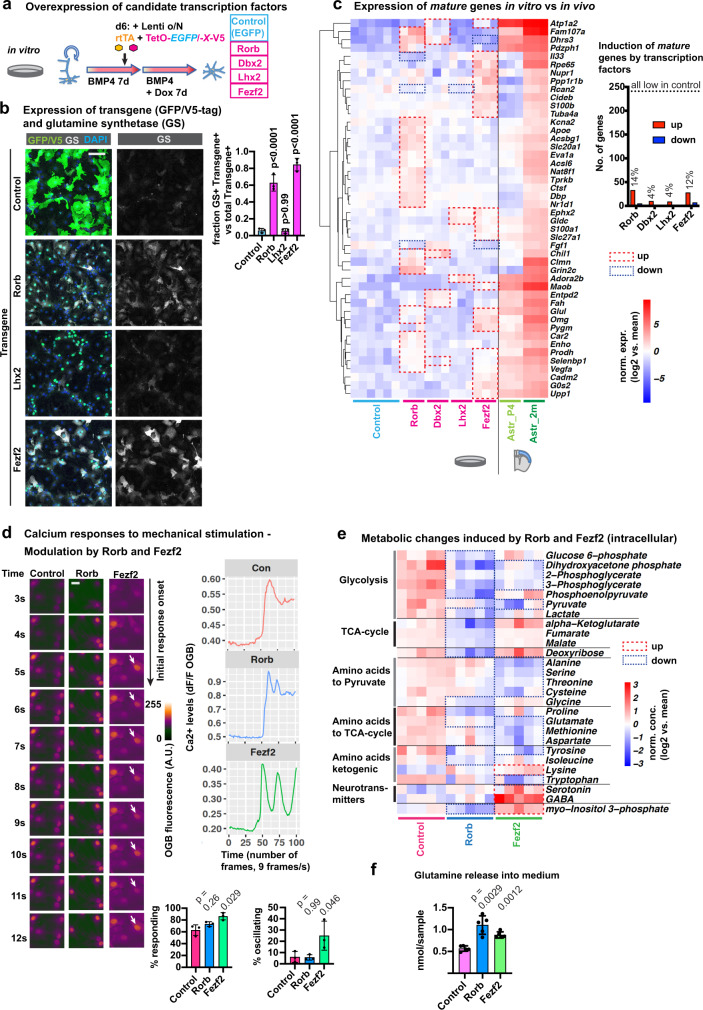
Different transcription factors control distinct aspects of astrocyte maturation. **a** Doxycycline-inducible expression of V5-tagged transcription factors (TFs) or EGFP as a control, in astrocyte cultures to assess the contribution of *Rorb*, *Dbx2*, *Lhx2* and *Fezf2* to astrocyte maturation. Cells are infected with the lentiviral vectors at day 6 of differentiation, followed by transgene induction with doxycycline from day 7 to day 14. **b** Immunolabeling for the transgene (EGFP or V5 tag) and the mature astrocyte enzyme glutamine synthetase (GS, gene *Glul*). **c** Expression of *mature astrocyte-specific* genes (from Fig. 2c) in cultured astrocytes and cortical astrocytes. Heatmap of selected genes. Barplot showing that subsets of *mature* genes with low expression in control cultures are induced by the transcription factors analysed. **d** Calcium responses induced by mechanical stimulation in Rorb- or Fezf2-overexpressing and control (rtTA expressing only) astrocytes, measured by time-lapse imaging with the calcium-sensitive dye OGB. **e**, **f** Metabolic changes induced by Rorb or Fezf2 in vitro, measured by gas chromatography coupled with mass spectrometry (GC-MS). Relative changes of intracellular metabolite levels (**e**), and glutamine release into the culture medium (**f**). Statistical analysis and data presentation: **b** scale bar 50 μm; shown: means ± SD; *n* = 3 biological replicates, one-way ANOVA (*F* = 117.4, *p* < 0.0001) with Tukey’s multiple comparisons test vs EGFP. **c** Differential genes: DESeq2 analysis, each *n* = 3; two-sided Wald test with Benjamini–Hochberg correction; significance threshold: adjusted *p*-value ≤ 0.05, absolute log2(fold change) ≥ 1. Shown are log2-transformed mean centred normalized expression values; **d** representative time-lapse series (pseudocoloured, scale bar 20 μm) and individual cell traces (displaying the change in fluorescence vs background (dF/F)), and barplots showing the fraction of responsive and oscillating cells (means ± SD, *n* = 3 biological replicates, repeated measures one-way ANOVA with Tukey’s multiple comparisons test; %responding: *F* = 7.711, *p* = 0.042; %oscillating: *F* = 7.878, *p* = 0.041); **e** Heatmap showing log2-normalised changes in metabolite levels relative to mean of each experiment (*n* = 5 independent experiments), Two-sided *t*-tests vs control with Benjamini–Hochberg correction for multiple testing; significant changes (padj < 0.05) are highlighted); **f** quantification of glutamine in samples of originally glutamine-free medium (means ± SD shown, *n* = 5 independent experiments, repeated measures one-way ANOVA with Tukey’s multiple comparisons test; *F* = 32.87, *p* = 0.0021); See also Supplementary Fig. 7, Supplementary Data 7, Supplementary Movies 1–3.

We next asked whether individual transcription factors that are induced during astrocyte maturation contribute to inducing the *mature* astrocyte phenotype, and whether the failure of some of these factors to be expressed in cultured astrocytes contributes to the lack of maturation of these cells. To address these questions, we performed a reconstitution assay in in vitro-differentiated astrocytes, whereby we used a lentiviral expression system to force expression in these cells of candidate transcription factors that normally remain expressed at low levels (Fig. 5a). We focussed on four candidates, the ROR protein Rorb, the HOX proteins Dbx2 and Lhx2 and the zinc finger protein Fezf2 (Fig. 4g). Efficiency of the delivery method was demonstrated by immunolabeling for the V5-tagged factors and for the mature astrocyte protein glutamine synthetase (GS/*Glul*), which was induced by both *Rorb* and *Fezf2* (Fig. 5b). We next performed a RNA-Seq analysis of the transduced cells to investigate the transcriptional activity of the candidate regulators, and compared these transcript profiles with those of postnatal and adult cortical astrocytes (Fig. 5c, Supplementary Fig. 7a–c, Supplementary Data 7). Both *Rorb* and *Fezf2* induced and repressed several hundred genes, whereas *Dbx2* and *Lhx2* regulated smaller gene sets (Supplementary Fig. 7a). Gene Ontology analysis revealed that the genesets induced by the transcription factors were enriched for terms related to adult astrocyte function such as ‘blood circulation’, ‘calcium homoeostasis’, ‘regulation of synaptic plasticity’ and ‘regulation of neurotransmitter levels’ (Supplementary Fig. 7a, Supplementary Data 7). The transcription factor-induced genes were significantly enriched for *mature astrocyte-specific* genes expressed only at low levels in control cultures (Supplementary Fig. 7b). *Rorb* alone induced 14% of the *mature* genes (33 out of 241 genes), including genes with well-established roles in astrocyte physiology such as *Glul* and *Apoe*, while *Fezf2* induced 12% (28 genes), *Dbx2* 4% (10 genes) and *Lhx2* 4% (9 genes) of this geneset (Fig. 5c, Supplementary Data 7). Interestingly, the genes regulated by the different transcription factors were largely non-overlapping, suggesting a modular control of the *mature* genes by distinct factors.

To assess whether the more mature transcriptional profiles result in improved functional maturation, we focussed on Rorb and Fezf2, which induced the most prominent transcriptional changes, and we analysed the functional consequences of the expression of these transcription factors on aspects of astrocyte physiology. As predicted by the GO analysis, imaging of cultures loaded with the calcium-sensitive dye OGB showed that Fezf2 altered calcium signalling in astrocytes (Fig. 5d, Supplementary Fig. 7d, Supplementary Movies 1–3). Fezf2 increased the propagation of calcium waves through adjacent astrocytes upon mechanical stimulation, which is a characteristic of mature gap-junction-coupled astrocyte networks^8^. Remarkably, Fezf2 also induced oscillatory calcium responses in a subset of cells, a pattern observed in astrocytes in acute brain slices^26^, further suggesting that Fezf2 might promote physiological astrocytic calcium responses.

Rorb and Fezf2 also induced several key metabolic enzymes required for mature astrocyte functions (Fig. 5b, c, Supplementary Data 7). We, therefore, assessed the metabolic state of in vitro-differentiated astrocytes expressing Rorb, Fezf2 or EGFP as control, using gas chromatography-mass spectrometry (GC-MS). We identified 47 metabolites in cell extracts and media supernatants, including amino acids and intermediates of glycolysis and the TCA cycle (Fig. 5e, f, Supplementary Fig. 7e, f, Supplementary Data 7). Interestingly, Rorb induced a depletion of intermediates of glycolysis and the TCA-cycle, while Fezf2 reduced intracellular amino acid levels and increased levels of the inhibitory gliotransmitter GABA, which is produced in astrocytes via *Maob*^27^, an enzyme induced by Fezf2 (Fig. 5c). While the release of metabolites into the culture medium was mostly unchanged or reduced by Rorb or Fezf2 (Supplementary Fig. 7e, f), both factors increased glutamine release (Fig. 5f), in line with their induction of glutamine synthetase (*Glul*), the key enzyme for the generation of this essential metabolite for the support of neurons^8,28^. Overall, these data strongly suggest that Rorb, Dbx2, Lhx2 and Fezf2 induce different sets of *mature* genes that control the maturation of different astrocytic properties such as their calcium responses and metabolic state, which are required by adult astrocytes to perform their functions.

### Transcription factors induce chromatin opening and cooperate to control maturation-regulated genes

**Fig. 6:**
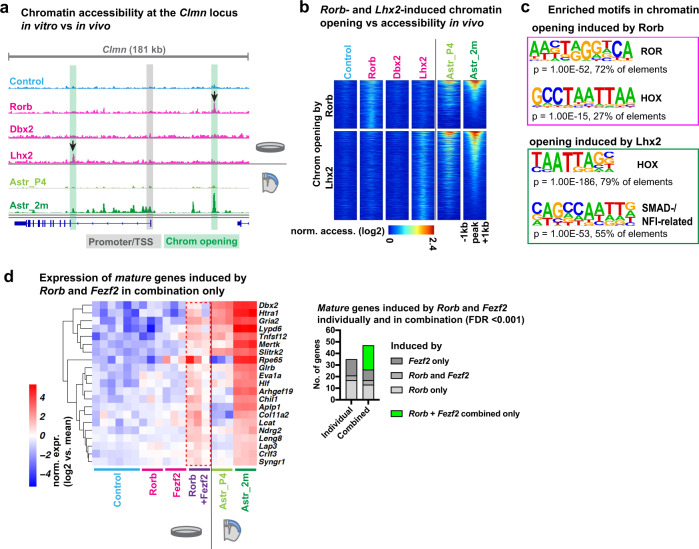
Transcription factors induce chromatin opening and cooperate to control maturation-regulated genes. **a** ATAC-Seq genome tracks showing chromatin accessibility peaks around the transcriptional start site (TSS) of the *mature* gene *Clmn* in cultured astrocytes expressing transcription factors and in brain astrocytes (at postnatal day 4 (Astr_P4), and 2 months of age (Astr_2m)). Note the highlighted peaks that are present in cultured astrocytes only when *Rorb* or *Lhx2* are expressed. **b** Heatmaps showing chromatin accessibility in cultured astrocytes around *Rorb*- and *Lhx2*-induced peaks and in brain astrocytes. **c** Transcription factor motifs enriched in *Rorb*- and *Lhx2*-induced peaks (de novo motif enrichment analysis). **d** Expression of *mature astrocyte-specific* genes induced by the combined expression of *Rorb* and *Fezf2*. Astrocytes co-infected with *Rorb-* and *Fezf2-inducing* viruses were analysed along with samples from Fig. 5. Statistical analysis and data presentation: **a** ATAC tracks represent merged reads of 3 replicates (4 for Astr_2m, 6 for Control). **b** Heatmaps show normalized ATAC read count of merged samples in 20 bp windows ±1  kb of peak centres. Differential peaks: DESeq2 analysis, each *n* = 3 (*n* = 4 for Astr_2m, *n* = 6 for Control); two-sided Wald test with Benjamini–Hochberg correction; significance threshold: adjusted *p*-value ≤ 0.05, absolute log2(fold change) ≥ 1. Shown are log2-transformed normalized expression values. **d** Differential genes: DESeq2 analysis, each *n* = 3 (*n* = 6 for Control); two-sided Wald test with Benjamini–Hochberg correction; significance threshold: adjusted *p*-value (FDR) ≤ 0.001, absolute log2(fold change) ≥ 1. Shown are log2-transformed mean centred, normalized expression values. See also Supplementary Fig. 8, Supplementary Data 8.

We next investigated the genomic mechanisms through which the identified transcription factors may control astrocyte maturation. To address whether the regulation of gene expression by ROR and HOX proteins in in vitro-differentiating astrocytes involves changes in chromatin accessibility, as suggested by our in vivo analyses (Fig. 3), we performed an ATAC-Seq analysis of cultured astrocytes expressing *Rorb*, *Dbx2* or *Lhx2*. Expression of *Rorb* and *Lhx2*, but not *Dbx2*, resulted in chromatin opening at the locus of the *mature astrocyte-specific* gene *Clmn* and at several hundreds other loci (Fig. 6a, Supplementary Fig. 8a). Many sites that acquired an ATAC-Seq peak upon transcription factor expression in vitro also had an ATAC-Seq peak in adult astrocytes in vivo (Fig. 6a, b). There was strong enrichment of ROR motifs in *Rorb*-induced ATAC peaks (72% of peaks) and of HOX motifs in *Lhx2*-induced peaks (79%) (Fig. 6c), suggesting that the changes in chromatin accessibility might largely be due to direct binding of the transcription factors to these elements. To further assess the contribution of direct transcription factor binding to chromatin remodelling, we compared the ATAC-Seq datasets to published ChIP-Seq datasets for Lhx2 in olfactory neurons^29^, Fezf2 in neurospheres^30^ and the transcription factor RORg (*Rorc*), which has a binding motif highly similar to that of Rorb-related^31^, in Th17 cells^32^. We reasoned that if these factors bind in the cell types used in these studies to loci that are accessible in astrocytes, these factors will be likely to also bind to these sites in astrocytes. This analysis indeed revealed a large overlap of the binding sites for these transcription factors with open chromatin in astrocytes (from ~20% of Lhx2 peaks to 75% of Fezf2 peaks) (Supplementary Data 8). Importantly, chromatin regions that gain accessibility in astrocytes upon overexpression of Lhx2 and Rorb were enriched in binding sites of Lhx2 and RORg, respectively, but not Fezf2 (Supplementary Fig. 8b, c), further supporting the hypothesis that direct binding of Rorb and Lhx2 contributes to chromatin opening during astrocyte maturation.

Since developmental genes are often regulated by multiple transcription factors acting cooperatively^33^, we examined a possible functional synergy between Rorb and Fezf2, by co-expressing the two genes in in vitro-differentiated astrocytes. RNA-Seq analysis revealed that co-expression of *Rorb* and *Fezf2* induced a number of additional *mature* genes that were not induced by either factor individually (Fig. 6f). When setting a low false discovery rate (FDR) threshold of 0.001, only 35 *mature* genes were induced by Rorb or Fezf2 separately (17 by Rorb, 14 by Fezf2, and 4 by both). The two factors together induced 47 genes, including 21 genes not induced by either individual factor (Fig. 6f). In combination, Rorb and Fezf2 also repressed several *immature* genes whose expression was maintained in in vitro-differentiated control astrocytes, including progenitor markers and genes supporting neuronal development, such as *Nes*, *Vim*, *Fabp7*, *Sparc*, *Marcks* and *Marcksl* (Supplementary Fig. 8d).

To assess potential mechanisms underlying this cooperative effect, we further compared our ATAC-Seq datasets with the published ChIP-Seq datasets for Fezf2 and RORg (Supplementary Fig. 8e, f). Focusing on putative regulatory elements associated with the *mature* genes uniquely induced by the combination of Rorb and Fezf2, we found a significant enrichment of *Fezf2* binding sites and several examples of regulatory elements bound by both *Fezf2* and RORg in the reference datasets. This analysis had limited statistical power due to the small size of the geneset, but broadening the analysis to the regulatory elements of all genes induced solely by the combination of Fezf2 and Rorb, showed a significant enrichment of putative regulatory elements containing RORg ChIP peaks as well as elements containing both Fezf2 and RORg ChIP peaks, i.e. elements in which Fezf2 and Rorb might physically interact (Supplementary Fig. 8f). Overall, this analysis suggests that Fezf2 and Rorb may cooperatively regulate astrocytic genes by binding together to the regulatory elements of these genes, including a subset of *mature astrocyte-specific* genes.

### Extrinsic signals promote the transcriptional and epigenetic maturation of astrocytes

**Fig. 7:**
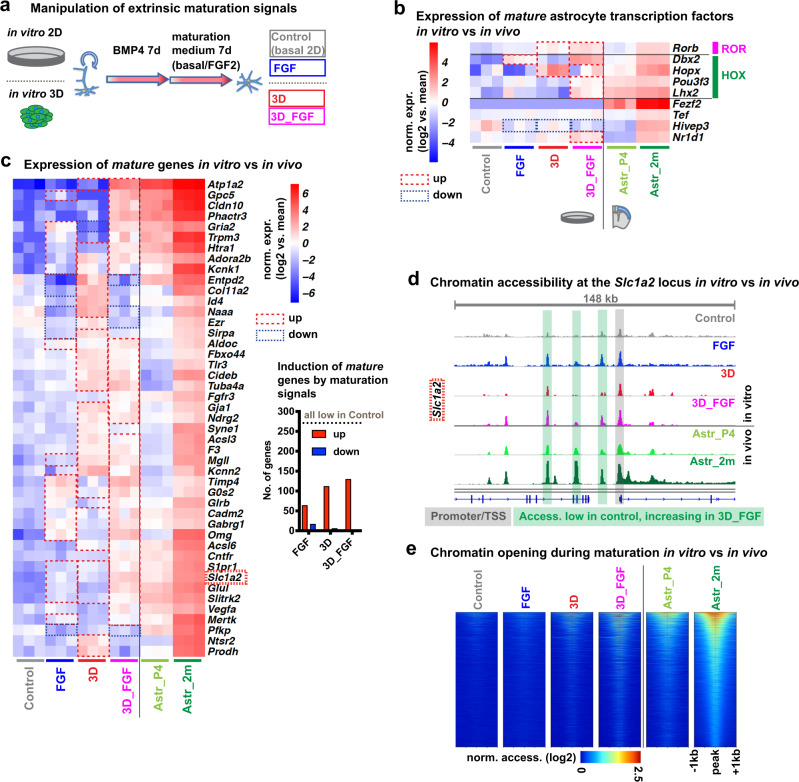
Extrinsic signals promote the transcriptional and epigenetic maturation of astrocytes. **a** Experimental design to assess the effects of extrinsic signals on astrocyte maturation. Initial differentiation with BMP4 is followed by 7 days in different maturation conditions: control (basal medium in conventional two-dimensional cultures) ±FGF2 (control/FGF) or in three-dimensional gel-embedded cultures (3D/3D_FGF). **b**, **c** Expression of *mature astrocyte-specific* transcription factors (**b**) and other genes (**c**) in cultured astrocytes and cortical astrocytes (at postnatal day 4 (Astr_P4), and 2 months of age (Astr_2m)). Heatmaps of selected genes. Barplot showing that subsets of *mature* genes with low expression in control cultures are induced in other culture conditions. **d** ATAC-Seq genome tracks showing chromatin accessibility peaks around the TSS of the *mature astrocyte-specific* gene *Slc1a2*, which is expressed at higher levels in astrocytes in 3D cultures in the presence of FGF2 than in astrocytes in control cultures (see (**c**)). **e** Heatmaps showing global chromatin accessibility around ATAC peaks that are induced during maturation of cortical astrocytes and in astrocytes in 3D cultures with FGF2, but are absent in control cultures (5499 peaks). Statistical analysis and data presentation: Differential genes/peaks: DESeq2 analysis, each n = 3 (*n* = 4 for Astr_2m); two-sided Wald test with Benjamini–Hochberg correction; significance threshold: adjusted *p*-value ≤ 0.05, absolute log2(fold change) ≥ 1. **b**, **c** Shown are log2-transformed mean centred, normalized expression values. **d** Tracks represent merged reads of 3 replicates (4 replicates for Astr_2m). **e** Heatmaps show normalised ATAC read count of merged samples in 20 bp windows ±1  kb of peak centres. Shown are log2-transformed normalized expression values. See also Supplementary Data 9.

The lack of expression of *Rorb*, *Dbx2*, *Lhx2* and *Fezf2* in in vitro-differentiated astrocytes suggested that these cultures were lacking a signalling environment that permits expression of these *mature astrocyte-specific* transcription factors. Previous studies have reported that cell–cell-contacts^14^ and FGF signalling^15^ could improve the functional maturation of cultured astrocytes. We, therefore, asked whether such conditions might stimulate astrocyte maturity by inducing the expression of *Rorb*, *Dbx2*, *Lhx2* or *Fezf2*. For this, we differentiated astrocytes for 7 days with BMP4 and cultured the cells for 7 additional days in control conditions (basal medium) or in the presence FGF2. We also differentiated astrocytes in a three-dimensional (3D) environment, in basal medium or in the presence of FGF2, to facilitate cell–cell-interactions (Fig. 7a, see ‘Methods’). RNA-Seq analysis of cultures in these different conditions revealed a significant increase in the expression of several of the *mature astrocyte-specific* transcription factors that remained expressed at low levels in control astrocyte cultures (Fig. 7b). In particular, addition of FGF2 in 3D cultures markedly induced the expression of *Rorb*, *Dbx2*, *Lhx2* and other factors (Fig. 7b) and increased the expression of a large number of other *mature astrocyte-specific* genes, including many important astrocyte effector genes such as *Slc1a2*, *Glul*, *Acsl6*, *Gja1*(Cx43) and *Mertk* (Fig. 7c, Supplementary Data 9). ATAC-Seq analysis showed that sites that were accessible in adult cortical astrocytes but not in control cultures, became accessible in FGF2-treated 3D cultures (e.g. at the *Slc1a2* locus, Fig. 7d, e). Together these findings suggest that ameliorating the signalling environment of in vitro-differentiating astrocytes can stimulate the expression of key transcriptional regulators which in turn promote astrocytic maturation, at least in part via chromatin remodelling at *mature astrocyte-specific* loci.

## Discussion

Astrocyte maturation, the last stage of astrocyte differentiation, takes place from early postnatal to young adult stages, when proliferating immature astrocytes cease to divide and aquire the full spectrum of astrocyte functions observed in the adult brain^1,16^. In this study, we have analysed in detail the transcriptional and chromatin changes that occur during maturation of astrocytes in the mouse forebrain. We found that defined extracellular signals can induce the expression of transcription factors associated with astrocytic maturation, which are otherwise lacking in cultured astrocytes. These transcription factors in turn induce chromatin remodelling and the expression of large numbers of genes associated with astrocyte maturity, which are organised in distinct regulatory modules activated by different transcription factors acting alone or in combination.

The global changes in gene expression occurring during astrocyte maturation have only recently begun to be investigated^11,13,34^, and their interpretation is complicated by two limitations: first, because of the phenotypic similarity of neural stem cells and astrocytes, it is difficult to experimentally isolate or even define cells that are considered immature astrocytes, as opposed to neural stem cells or glial progenitors. Second, some of the papers investigating astrocyte maturation are based on in vitro models that do not fully recapitulate in vivo maturation^11,13^. Nevertheless, these recent studies have revealed major transcriptional changes occurring during astrocyte maturation, in agreement with the findings in this study. However, single-cell RNA-Seq analysis allowed us to analyse gene expression changes with greater resolution than in previous studies. We propose in particular that proliferating immature astrocyte progenitors, which based on their expression profiles (expression of Olig1/2 and low levels of astrocyte markers) likely correspond to the “intermediate glial progenitor cells” (iGCs) described in a recent study^35^, progress to a fully mature state through at least two intermediate stages in which astrocytes have exited the cell cycle but remain immature.

By integrating ATAC-Seq and RNA-Seq data, we identified dynamically accessible putative regulatory genomic elements present either in immature or mature astrocytes in vivo, which suggests that chromatin remodelling has an important role in the transcriptional changes taking place during astrocyte maturation. Motif enrichment suggests that transcription factors acting downstream of extracellular signals maintain immature astrocyte-specific enhancers in an open state. These factors include ETS factors, whose activity is regulated by receptor tyrosine kinase receptor signalling^36,37^, the Wnt pathways effectors LEF/TCF^38^, and NF-κB acting downstream of inflammatory signals^39,40^. Thus the limited availability of these developmental signals in the adult brain might lead to the loss of expression of *immature astrocyte* genes involved in the unique properties of immature astrocytes, including perhaps their capacity to reactivate NSC programmes and become proliferative and neurogenic. In the future it will be interesting to determine whether reactivating these developmental pathways could restore plasticity to mature astrocytes. Conversely, we found that the upegulation of HOX transcription factors, including *Dbx2* and *Lhx2*, and ROR transcription factors, including *Rorb*, might result in the opening of enhancers required for the expression of *mature astrocyte* genes.

To functionally dissect the mechanisms underlying astrocyte maturation, we used an in vitro model of differentiation of cultured NSCs into astrocytes in the presence of BMP4^9,10^. We found that astrocytes differentiated with this protocol remained partially immature, similar to astrocytes differentiated with CNTF^11^ and cultured primary astrocytes^17^. This finding highlights the difficulty of studying astrocyte maturation and physiology in culture, a serious handicap for this field^12,15,17^. This lack of maturation has recently been ascribed to the absence of relevant extrinsic signals, including growth factors and contacts with neurons and other astrocytes^14,15,17^. However, a limitation of these studies is that the maturity of in vitro-differentiated astrocytes was not compared with that of mature brain astrocytes. Here we found that three-dimensional cultures, which allow more extensive cell–cell-contacts, showed improved, though not complete, astrocyte maturity, particularly in combination with FGF2. These findings show that our approach of comparing the molecular profile of in vitro-differentiated astrocytes with that of astrocytes acutely isolated from the brain is a powerful strategy to further improve differentiation protocols for cultured astrocytes.

While the incomplete maturation of astrocytes in vitro limits their suitability as models to study astrocyte function, it provided an excellent assay to investigate the mechanisms that promote maturation. By reconstituting the expression of transcription factors that are induced in astrocytes maturing in vivo but are missing from cultured astrocytes, we could demonstrate a maturation-promoting activity of four factors, *Rorb*, *Dbx2*, *Lhx2* and *Fezf2*. These factors have mainly been studied for their roles in neuronal subtype specification^41–44^. These functions in neurons seem to be mediated at least partially by cross-repression of alternative neuronal subtype programmes, suggesting that their expression in astrocytes might contribute to the global suppression of neuronal programmes. Moreover, *Dbx2* and *Fezf2* have also been shown to promote NSC quiescence^45,46^, suggesting that these factors might contribute to suppressing the proliferative capacity of mature astrocytes.

Interestingly, in astrocytes the four factors induced largely non-overlapping sets of astrocyte effector genes, suggesting that the astrocyte maturation programme is regulated in a modular manner. This is different from cell fate specification and early differentiation steps, where individual master regulators trigger a highly coordinated cell intrinsic differentiation programme. For example, NFIA can trigger astrocyte differentiation of human NSCs^47^. In contrast, our results suggest that maturation is controlled by a large number of transcription factors that induce distinct astrocytic effector subprogrammes. These differences may be explained by different regulatory requirements. Early differentiation establishes the fundamental properties of a cell type largely independently of extrinsic conditions, and therefore relies mostly on cell intrinsic master regulators and downstream transcription factor networks. In contrast, maturation programmes shape the cellular effector functions and must therefore be adapted to the changing requirements of the organism, particularly for a highly plastic and multifunctional cell type such as astrocytes. This adaptability is best maintained if different functions are controlled independently by different transcription factors that are responsive to extrinsic factors that relay various physiological requirements. This modular maturation model is supported by our results showing that astrocytes respond to FGF2 and three-dimensional culture by inducing the transcription factors *Rorb*, *Dbx2* and *Lhx2*, which in turn induce different sets of mature astrocyte genes, and also by our findings that Rorb and Fezf2 have distinct, independent effects on calcium signalling and metabolism in cultured astrocytes. It remains to be investigated to what extent this signal-dependent modular organisation contributes to the increasingly recognised local and regional molecular heterogeneity of astrocytes^48–50^, as well as to what extent this model can be generalised to other cell types. Interestingly, a similar model has also been discussed for the maturation of T lymphocyte subtypes, which are determined by a regulatory network involving cytokines as extrinsic signals and a spectrum of transcription factors rather than individual master regulators^51^.

Importantly, we also found that the maturation-associated transcription factors do not act fully independently but cooperate on several levels. First, they induce distinct sets of effector genes that may regulate the same astrocyte functions. For example, *Apoe* (induced by *Rorb*), *S100b* (induced by *Fezf2*), and *Atp1a2* (induced by *Dbx2* and *Fezf2*) have all been reported to regulate intracellular calcium levels in astrocytes^52–54^. Second, the induction of chromatin remodelling by *Rorb* and *Lhx2* might be a prerequisite for other factors to promote the expression of astrocytic effector genes. In particular *Lhx2* induces chromatin opening at a large number of sites but regulates only few genes, suggesting that *Lhx2*-dependent chromatin opening may “bookmark” genes for future induction by other factors, as has been recently reported for *C/EBPa* and *HNF4a* in liver development^55^. Such a mechanism might contribute to the observed cooperativity of *Rorb* and *Fezf2*, which together induced the expression of many more genes than were induced by each factor separately. One potential explanation for this cooperative activity might be that *Rorb* opens enhancers that are subsequently bound and activated by *Fezf2*. Alternatively, the two factors might bind simultaneously to the same or different enhancers of the same gene^33^.

Additional research is required to fully determine the functions of the transcription factors investigated here, in promoting specific aspects of the mature phenotype of astrocytes. *Lhx2* has been shown to contribute to the differentiation of tanycytes and the maintenance of the mature state of Müller glia, two astrocyte-like cell types found in the hypothalamus and the retina, respectively^56,57^, while *Rorb*, *Dbx2* and *Fezf2* have not been previously implicated in the development of glial cells. Our results suggest functions for *Rorb* and *Fezf2* in astrocytes, in particular a role in the rewiring of glucose or amino acid metabolism to increase the production of glutamine, which serves as a precursor for the major excitatory and inhibitory neurotransmitters glutamate and GABA^8,28,58^. Furthermore, we show that *Fezf2* can modulate calcium signalling and induce the production of the inhibitory gliotransmitter GABA, which is required for the tonic inhibition of neurons by astrocytes^27^. Through these functions, *Rorb* and *Fezf2* might be critical for the maintenance of normal neuronal excitability. Interestingly, supporting this hypothesis, pathogenic mutations in *RORB* have been identified in genetic forms of epilepsy^59^. Since astrocyte dysfunction is considered a key pathological mechanism in epilepsy^60^, these *RORB* mutations may act by blocking the induction of mature astrocyte genes protecting from epileptogenesis^60^, such as the *Rorb* target *Glul*, whose deletion in astrocytes causes seizures and neurodegeneration in mice^28^, or the glutamate transporter *Slc1a2*, which has been shown to be mutated in epileptic encephalopathies^61^.

In conclusion, our study has identified extracellular signals and transcription factors that drive distinct parts of the astrocyte maturation programme through chromatin and transcriptional reorganisation, and thereby regulate important aspects of astrocyte functions in brain homoeostasis. With this, our study opens up directions of research that may contribute to better understand and ultimately treat neurological disorders involving astrocyte dysfunction.

## Methods

### Mouse models

Animal care and procedures were performed in accordance with the guidelines of the Francis Crick Institute, as well as national guidelines and laws, under the UK Home Office Licence PPL PB04755CC. Mice were housed in standard IVC cages with ad libitum access to food and water, under a 12/12 h light/dark cycle, at a temperature of 19–21 °C and a humidity of 45–65%. Brain tissue was obtained from male C57BL/6Jax mice at postnatal day 3 (P3), P4, or at an age of 6–10 weeks (2 m) or 12–14 weeks (3 m).

### Primary neural stem/progenitor cells

Primary neural stem/progenitor cells (NSCs) were derived from male mice of a mixed MF1-derived background. Three batches were used in this study: batch X6 (from adult subventricular zone tissue, age 8 weeks), batches X8 and X9 (from P3 postnatal cortical grey matter tissue).

Tissue was dissociated with the ‘Neural Tissue Dissociation Kit (P)’ (Miltenyi, 130-092-628) and cell preparations were initially expanded for 1–2 passages as neurospheres in *basal medium* ((DMEM/F-12 + Glutamax (Invitrogen 31331-093) + 1× N2 supplement (R&D Systems, AR009) + 1× penicillin–streptomycin (ThermoFischer Scientific, 15140)) supplemented with 20 ng/ml EGF (Preprotech, 315-09-100) + 20 ng/ml FGF2 (Peprotech, 450-33) + 5 μg/mL Heparin (Sigma, H3393-50KU).

Subsequently cells were further expanded as adherent cultures in *basal medium* supplemented with EGF + FGF2 + Heparin (as above), + 2 μg/ml Laminin (Sigma, L2020) (*EGF/FGF2-medium*), on plates pre-coated for > 1 h with EGF/FGF2 medium. For passaging, cells were detached with Accutase (Sigma, A6964). Cells were maintained at 37 °C, 5% CO_2_ and used for experiments at total passage 7–25.

### MACS-based astrocyte purification

Astrocytes were purified using the “Anti-ACSA-2 MicroBead Kit, mouse” (Miltenyi, 130-097-678) with the protocol recommended by the manufacturer for adult astrocytes.

Brains of two animals to five per preparation were cut into coronal slices and cortical grey matter or striatal tissue (excluding the subventricular zone) was dissected. The tissue was cut to small pieces and dissociated with the ‘Neural Tissue Dissociation Kit (P)’ (Miltenyi, 130-092-628), incubating for a total of 30 min at 37 °C under gentle agitation. Gentle mechanical dissociation was applied after addition of DNase at 15 min, and more thorough dissociation after 25 min (using fire-polished pipettes). After removal of remaining incompletely dissociated tissue pieces using a 70 μm cell strainer, the enzymatic dissociation reaction was stopped with calcium-containing HBSS buffer. All subsequent steps were performed on ice or at 4 °C with pre-cooled reagents.

The cells were collected by centrifugation (300 × *g*, 10 min, 4 °C) and resuspended in 6.2 ml DPBS + 1.8 ml ‘Debris removal solution’ (Miltenyi 130-109-398). The suspension was overlaid with 4 ml DPBS, followed by centrifugation at 3000 × *g*, 10 min, 4 °C. The upper two gradient layers were removed (retaining ~1 ml), 10 ml DPBS added, and the cells collected by centrifugation at 1000 × *g*, 10 min, 4 °C. Cells were incubated in ‘Red blood cell removal solution’ (Miltenyi 130-094-183) for 10 min. After addition of 10 ml PB buffer (DPBS + 0.5% BSA), cells were collected by centrifugation (300 × *g*, 10 min, 4 °C), resuspended in 80 μl PB + 10 μl Fc-Block from the ACSA2-Kit, and incubated for 10 min. 10 μl magnetic-bead conjugated Anti-ACSA2-antibody was added for 15 min. Cells were collected by centrifugation and resuspended in 500 μl PB. The suspension was passed through a 70 μm cell strainer and a pre-equilibrated MS column (Miltenyi 130-042-201) in an magnetic separator (OctoMACS, Miltenyi 130-042-109). The column was washed twice with PB before the column was removed from the separator to wash out the purified astrocyte preparation with PB.

### Astrocyte differentiation in vitro (2D cultures)

Neural progenitors maintained in vitro at passage 7–25 were plated in a basal medium consisting of DMEM/F-12 supplemented with Glutamax (Invitrogen 31331-093), N2 supplement (R&D Systems, AR009) and penicillin–streptomycin (Thermo Fisher Scientific, 15140), additionally supplemented with 20 ng/ml EGF (Preprotech, 315-09-100), 20 ng/ml FGF2 (Peprotech, 450-33), 5 μg/mL Heparin (Sigma, H3393-50KU) and 2 ug/ml laminin (Sigma, L2020) (refered to as EGF/FGF2-medium). Progenitors were plated at a density of 40,000 cells/cm^2^ on cell culture dishes coated for >1 h in EGF/FGF2-medium. Coverslips for immunolabelling were pre-coated before for >1 h with 10 μg/ml laminin. After 1 day the medium was changed to BMP4-medium (the basal medium described above, supplemented with 2 μg/ml laminin and 20 ng/ml BMP4 (Biolegend, Cat. No. 595302)), which was renewed every 2–3 days for 14 days or as indicated in specific figures. For the study of extrinsic maturation signals, after 7 days the BMP4-medium was replaced by basal medium supplemented with 2 μg/ml laminin) or FGF2-medium (basal medium supplemented with 2 μg/ml laminin and 20 ng/ml FGF2), which also was changed every 2–3 days for further 7 days. All cultured cells were maintained at 37 °C, 5% CO_2_. At the end of the differentiation protocol, cells were either fixed with 4% PFA for 15 min for immunofluorescence, lysed with Trizol for RNA extraction, or detached with accutase (Sigma, A6964).

Sets of experiments with cultures differentiated independently at different timepoints using different neural progenitor preparations were considered as biological replicates.

### Astrocyte differentiation in vitro in 3D cultures

For 3D cultures, neural progenitors were initially cultured for 7 days as neurospheres in suspension in *EGF/FGF2-medium* without laminin (as described for 2D cultures) at a density of 120,000 cells/ml. Spheres were collected by centrifugation (100 × *g*, 3 min) and plated at a density equivalent of 100,000 originally plated cells/well in 24-wells. Cells were plated in a basal membrane extract (BME) gel of EGF/FGF2-medium supplemented with 20% “Cultrex PathClear 3-D Culture Matrix RGF BME” (Bio-Techne, 3445-010-01), in wells pre-coated with EGF/FGF2 + 20% BME. Cells were differentiated with BMP4-medium, followed by basal medium or FGF2-medium (see the previous section; each supplemented with 2% BME) as described for 2D cultures.

As 3D conditions may result in more heterogenous differentiation, e.g. due to residual growth factors in the 3D gel, or limited diffusion of growth factors into the gel, MACS purification was performed to remove potentially occurring contaminating cells. For this, the gel and the embedded spheres were dissociated by addition of Dispase (Life Technologies, 17105041, final concentration 3 mg/ml) to the culture medium for 1 h, and gentle mechanical dissociation. Cells were then collected by centrifugation, resuspended in DPBS + 0.5% BSA, blocked with Fc-Block reagent, labelled with ACSA2 antibodies and purified on MACS columns as described above for the purification of astrocytes from brain tissue.

### Lentiviral expression of candidate transcription factors in a doxycycline-regulated Tet-on system

*Rorb*, *Dbx2*, *Lhx2*, and *Fezf2* open reading frame (ORF) constructs from Origene (MR225771, MR215327, MR206390, MR207270) were re-cloned into a modified version of the TetO-FUW-EGFP lentiviral expression plasmid (Addgene #30130)^62^. In this vector we had replaced *EGFP* by *Nr1d1* with a C-terminal V5 tag, and added additional restriction sites to facilitate exchange of Nr1d1 with other ORFs. We then replaced *Nr1d1* with *Rorb*, *Dbx2*, *Lhx2* or *Fezf2* to generate V5-tagged doxycycline-inducible lentiviral expression vectors for these transcription factors.

For lentivirus production, these plasmids, the TetO-FUW-EGFP control plasmid, or the FUW-M2rtTA driver construct (Addgene #20342)^63^ were transfected into 293FT cells, together with the third-generation lentiviral packaging/envelop plasmids pMD2.G (Addgene #12259), pRSV-rev (Addgene #12253) and pMDLg/pRRE (Addgene #12251)^64^. Three days after transfection, virus particles were collected by ultracentrifugation of the medium. Virus particles were resuspended as highly concentrated virus stock in DPBS, which was stored in aliquots at −80C. Virus titers were determined in BMP4-differentiated astrocytes at day 3, which were infected over-night infection of days with the FUW-M2rtTA and any TetO-FUW virus. Transgene expression was induced by addition of doxycycline (2ug/ml) for 2 days, and determined by immunostaining for EGFP or the V5 tag of the transcription factors.

For the transcription factor overexpression studies, FUW-M2rtTA was transduced along with TetO-FUW-EGFP or any of the transcription factor expression constructs, by over-night infection of astrocytes at a multiplicity of infection (MOI) of 3, at day 6 of the differentiation protocol. After infection, the transgenes were induced by addition of doxycycline to the differentiation medium (BMP4-medium) for 7 days with renewal every 2–3 days.

### Immunofluorescence stainings and microscopy

For stainings of brain sections, 40 μm coronal sections were obtained using a vibratome (Leica, Deerfield, IL) from mice that had been transcardially perfused with PBS followed by 4% paraformaldehyde in PBS (10 min), postfixed for 16–24 h and then stored in PBS with 0.02% sodium azide. Before stainings, antigen retrieval was performed by incubating the sections in sodium citrate buffer (10 mM, pH 6.0) at 95 °C for 10 min, and sections were permeabilised with 1% Triton in PBS for at least 90 min. Cultured cells on glass coverslips were fixed with 4% PFA and stored in PBS at 4 °C and before the staining washed with PBS + Triton (0.1%).

Following primary antibodies were used: Rabbit anti-BLBP (Millipore, ABN14, dilution 1:400), Rabbit anti-Cx30 (Thermo Scientific, 71–2200, 1:400), Rat anti-GFAP (Thermo Fisher, Cat# 13–0300, 1:500), Goat anti-GFP (Abcam, Cat# ab6673, 1:500), Guineapig anti-GLT-1 (Millipore, AB1783, 1:1000), Mouse anti-GS (BD Biosciences, Cat# 610517, 1:300), Rabbit anti-Id1 (Biocheck, BCH-1/#37-2, 1:100), Rb anti-Id3 (Biocheck, BCH-4/#17-3, 1:100), Mouse anti-Ki67 (BD Biosciences, Cat# 550609, 1:200), Rabbit anti-Ki67 (Leica Biosystems, Cat# NCL-Ki67p, 1:200), Mouse anti-Nes (Millipore, MAB353, 1:200), Mouse anti-S100b (Sigma, S2657, 1:200), Goat anti-Sox9 (R&D, AF3075, 1:200), and Goat anti-V5 (Bethyl Biolabs, Cat# A190-119A, 1:200). The secondary antibodies used are (all raised in donkey, dilution 1:500): anti-Goat-Alexa Fluor 488 (Thermo Fisher, A11055), anti-Rat-Alexa Fluor 488 (Thermo Fisher, A21208), anti-Rabbit-Alexa Fluor 568 (Thermo Fisher, A10042), anti-Guineapig-Alexa Fluor 647 (Jackson ImmunoResearch 706-605-148), and anti-Mouse-Alexa Fluor 647 (Jackson ImmunoResearch 715-606-151).

Coverslips or brain sections were then incubated with *blocking solution* (10% normal donkey serum in PBS/Triton) for 1 h at room temperature. Subsequently, coverslips/sections were incubated over night at 4 °C with primary antibodies in *blocking solution*, washed three times with PBS/Triton, and incubated with secondary antibodies in blocking solution for 2 h at room temperature. After one wash with PBS/Triton and 2 washes with PBS, the coverslips/sections were stained with DAPI (1 μg/ml) in 0.5x PBS for 15–30 min, and washed again with 0.5× PBS before mounting. Images were acquired with a Leica SP5 confocal microscope (with the LAS AF 2.7.9723 image acquisition software) with a ×20 objective, with z-steps of 1 μm, for tissue sections for 10 z-layers or for cultured cells through the whole-cell monolayer (approx. 8–15 z-layers).

### Ca^2+^ Imaging

Astrocyte cultures were incubated with 10 μM Oregon Green Bapta 1 (OGB, Invitrogen), diluted in culture medium, for 45 min at 37 °C in humidified atmosphere. After the incubation time, cells were placed in a recording chamber mounted on a fluorescent microscope (SliceScope Pro 6000 System, Scientifica) and perfused with artificial cerebrospinal fluid (ACSF) (in mM): NaCl, 125; KCl, 2.5; NaHCO_3_, 25; NaH_2_PO_4_, 1.25; CaCl_2_, 2; MgCl_2_, 1 and glucose, 12, saturated with 5% CO_2_ and 95 % O_2_ (pH 7.4, 30–32 °C). Cells were visualized with a ×40 (0.7 NA) objective and movies were acquired with a frequency of 9 Hz with a Hamamatsu Orca-Flash 4.0 camera and Micro-Manager 1.4 software. During acquisition, Ca^2+^ waves generation was induced by producing mechanical stimulation of a single astrocyte positioned in the centre of field of view with a glass pipette (resistance of 5–10 MΩ, which corresponds to a tip opening of 1–2 mm). For quantification, regions of interest (ROIs) were defined in all cells in the field of view with a recognizable nucleus (between 25 and 45 cells/movie). Three independent experiments were performed. From each of them, two biological replicates were analysed with a total of 6–8 videos acquired per condition. Intensity measurements for ROIs are expressed as the change of fluorescence relative to background fluorescence (ΔF/F0) and were calculated with ImageJ (ROI manager).

### Single-cell RNA-sequencing and analysis

For single cell mRNA-Seq, single cell suspensions of striatal astrocytes were prepared by MACS as above. Samples were prepared using the 10x Chromium Next GEM Single Cell 3ʹ v3.1 kit. For each sample, cells were quantified using an automated cell counter and viability assessed via trypan blue staining. Where possible, approximately 10,000 cells were loaded into the 10x Genomics Chromium Controller. Following GEM recovery, cDNA was amplified and final libraries prepared according to the manufacturer’s instructions. Finished libraries were quantified using the Qubit (Thermofisher) and TapeStation (Agilent) and pooled for sequencing with the objective of achieving 50,000 reads per cell. Sequencing was carried out on the HiSeq 4000 (Illumina), with 28 cycles for sequencing read 1, 8 cycles for index read 1, and 98 cycles for sequencing read 2.

The resulting data was demultiplexed using the 10x cellranger mkfastq function according to the manufactuer’s instructions. Reads from individual samples were mapped to the mouse genome (mm10) and quantified with the cell ranger count function (v3.0.2, option --expect-cells = 3500). The generated count matrices were imported into Seurat (v3.2.2)^65^ and aggregated using the Seurat ‘merge’ function. Low quality cells with more than 10% mitochondrial transcripts or less than 500 detected genes were removed. Expression values were normalised with the ‘SCTransform’ function, followed by dimension reduction with ‘RunPCA’, and generation of a UMAP plot using ‘RunUMAP’. ‘FindNeighbours’, followed by ‘FindClusters(…, resolution = 0.6)’ was used to identify cell clusters. ‘Dimplot(…, group.by = ‘orig.ident’)’ was used to analyse contribution of different samples to the clusters. ‘FeaturePlot’ and ‘DotPlot’ functions were used to plot expression of marker genes to identify the cell type identity of the clusters. The clusters of Sox9+ astroglial cells (clusters 0, 1, 3, 4, 5, 8, 9, 11, 14, 15, 19) were isolated and re-clustered as above (FindClusters(…, resolution = 0.7)).

To infer lineage relationships and a pseudotime order of cells, Slingshot^66^ (version 1.6.1) was used on the reclustered dataset with the parameters slingshot(…, reducedDim = ‘PCA’, clusterLabels = sce$seurat_clusters, start.clus = 6, approx_points = 100).

To identify genes with dynamic expression along pseudotime, the fitGAM() and associationTest() functions from the ‘tradeSeq’ R package^67^ (version 1.2.01) were used. To filter out genes with low levels of pseudotemporal change and group genes with similar expression patterns, cells were grouped into 20 pseudotime bins encompassing equal pseudotime ranges, and the mean of SCT-normalised expression was calculated for each bin with more than 10 cells (bins 2–19). After further normalisation by centering on the mean expression in all pseudotime bins, genes with minimal expression changes (maximum deviation from the mean of all bins <0.2) were removed. The normalised expression values for the remaining, dynamically regulated, genes were plotted and grouped by hierarchical clustering into 50 initial modules using the ‘pheatmap’ R package (version 1.0.12), with the parameters ‘pheatmap(…, clustering_distance_rows = ‘euclidean’, clustering_method = ‘ward.D2’, cutree_rows = 50)’. Gene modules with similar profiles were then manually merged to the final 6 modules shown in Fig. 1f.

Gene Ontology analysis was performed using the R package ‘clusterProfiler’ (version 3.16.1)^68^, using the function ‘enrichGO(…, OrgDb = org.Mm.eg.db, keyType = ‘SYMBOL’, ont = ‘BP’, pAdjustMethod = ‘BH’, pvalueCutoff = 0.01, qvalueCutoff = 0.05)’.

For the comparison of the astrocyte clusters with the reference dataset from Zeisel et al.^20^ (Figs. S1D, S2C), the ‘l6_r3_astroependymal_cells.loom’ subset of the reference atlas was downloaded from http://loom.linnarssonlab.org/, imported into Seurat using the loomR package (version 0.2.1.9000). Cells with ‘TaxonomyRank4 = = ‘Astrocytes’ ’ were integrated with the re-clustered astrocyte subset of the striatal scRNA-Seq analysis using the Seurat functions SelectIntegrationFeatures(…, nfeatures = 3000), FindIntegrationAnchors) and IntegrateData() using SCT-normalised expression data. The merged dataset was then clustered as described above, using the ‘integrated’ assay and the FindClusters() function with FindClusters(…, resolution = 0.8). To compare gene expression between the different datasets, the mean SCT-normalised expression for each gene in each of the original clusters was calculated and centered on the mean of all clusters. Expression values were then plotted using the pheatmap() function, allowing reordering of genes and clusters by hierarchical clustering.

### Bulk RNA sequencing

RNA was extracted using Trizol, from frozen pellets of MACS-purified astrocytes, or by direct lysis of cultured cells on culture dishes. The ‘Direct-zol RNA MiniPrep Kit’ (Zymo Research, R2052) was used for purification of the extracted RNA, according to manufacturer’s instructions, including the optional Dnase digestion step. The extracted RNA was quantified using the GloMax (Promega) and TapeStation (Agilent) using high sensitivity RNA ScreenTapes, or high sensivity BioAnalyser (Agilent). RNA was prepared into cDNA using the NuGEN Ovation RNA-Seq System V2 kit (Tecan). Following cDNA generation, cDNA was fragmented to an average length of 300 bp using the Covaris E220 focused ultrasonicator (Covaris). Final libraries were then prepared using the NuGEN Ovation Ultralow Library System V2 (Tecan) according to the manufacturer’s instructions. Final libraries were quantified using the TapeStation with D1000 ScreenTapes (Agilent), pooled to 4 nM and sequencing on the HiSeq 4000 system (Illumina) with at least 75 bp single ended reads. The resulting data were demultiplexed using the Illumina bcl2fastq program.

### RNA sequencing analysis

Reads from RNA-Seq fastq files were aligned to the mm10 genome and quantified using the crickbabs/BABS-RNASeq nextflow pipeline (https://github.com/crickbabs/BABS-RNASeq; git commit id: 335ce47db079d6cc2a7f82f4b762620c4f7f27e2). The pipeline was run using nextflow version 0.30.2^69^ with the command ‘nextflow run main.nf -params-file params.yml’.

After alignment and transcript quantification, all downstream analysis was performed with R. Raw count normalisation and differential expression analysis was performed using the Bioconductor ‘DESeq2’ package (v1.24.0)^70^. Lowly expressed genes (maximal normalised expression <10) were removed, and genes with an adjusted *p*-value (padj ≤ 0.05) and at least 2-fold change in expression (abs(log2FC) ≥ 1) were considered to be differentially expressed. Relative expression heatmaps were generated using the ‘pheatmap’ R package (v1.0.12) and plotted as log2 transformed DESeq2 normalised expression values, centered on the mean expression of each gene over all plotted samples. Gene Ontology analysis was performed using the R package ‘clusterProfiler’^68^, as described for scRNA-Seq.

Known or predicted transcription factors from the Fantom5 transcription factor database were classified as transcription factors in this study (http://fantom.gsc.riken.jp/ 5/sstar/Browse_Transcription_Factors_mm9, downloaded 27/10/2018). Published in vitro astrocyte gene expression data from other models^11,17^ was re-analysed with the nextflow pipeline above, then merged with the datasets of this study and analysed as above.

### ATAC sequencing

ATAC sequencing libraries were prepared using the OMNI-ATAC protocol^71^ with minor modifications:

25,000 MACS-purified astrocytes or dissociated cultured cells were collected by centrifugation (300 × *g*, 5 min, 4 °C), and permeabilized for 3 min on ice in 50 μl ATAC-RSB buffer (Tris/HCl (pH 7.4) 10 mM, NaCl 10 mM, MgCl_2_ 3 mM) containing 0.1% NP-40 (IGEPAL CA-630), 0.1% Tween-20 and 0.01% digitonin. 1 ml ATAC-RSB buffer containing 0.1% Tween-20 was added and the nuclei were recovered by centrifugation (500 × *g*, 10 min, 4 °C). The pellet was resuspended in 25 μl of transposase reaction mix (12.5 μl 2× TD buffer and 1.25 μl Tn5 transposase (Illumina Cat #FC-121-1030), 8.25 μl PBS, 2.5 μl water, 0.1% Tween-20, 0.01% digitonin), and incubated on a rotational shaker (1000 rpm) for 30 min at 37 °C to fragment and tag accessible chromatin. DNA was then purified using the ‘MinElute PCR purification kit’ (Qiagen, Cat no. 28004) and eluted in 20 μl EB buffer (Qiagen). 5 μl DNA were amplified by PCR in 20 μl reactions using NEBNext HiFi 2× PCR Master Mix (New England Biolabs, M0541) and barcoded primers described previously^22^ (see Supplementary Table 1) for 12 cycles: (1) 5 min 72 °C, (2) 30 s 98 °C, (3) repeat 12×: 10 s 98 °C, 30 s 63 °C, 60 s 72 °C. The amplified libraries were purified using SPRIselect beads (Beckman Coulter, Cat# B23319), ratio sample: beads 1:1.8) to remove small DNA fragments like primers and primer dimers. For all steps until and including the library amplification step, ‘Nonstick, RNase-free Microfuge Tubes’ (Thermo Fisher, AM12350 or 10676825) were used.

ATAC libraries were quantified using the BioAnalyser (Agilent) and pooled to 4 nM for sequencing on the HiSeq 2500 or HiSeq 4000 system (Illumina). With either instrument, libraries were sequenced with 100 bp paired end reads. Following sequencing, the data were demultiplexed using the bcl2fastq program (Illumina).

### ATAC sequencing analysis

Reads from ATAC-Seq fastq files were aligned to the mm10 genome, peaks mapped and quantified using the crickbabs/BABS-ATACSeqPE nextflow pipeline (https://github.com/ crickbabs/BABS-ATACSeqPE; git commit id: 22edccf72855d42e6692a27385cf50666c8f391c; now superseded; a newer version is available as part of the nf-core project: nf-core/atacseq pipeline (10.5281/zenodo.2634132^72^). The pipeline was run using nextflow version 0.30.2^69^ with the command ‘nextflow run main.nf --design../design.csv --genome mm10 -profile conda --outdir../results/’.

All downstream analysis was performed with R, except transcription factor binding motif enrichment, which was performed using the Homer software^73^.

After initial peak calling, annotation and quantification on the merged dataset, the functional peak annotation and classification was extended using multiple published datasets (see also Fig. S2B). First, promoters identified by the original pipeline and peaks overlapping with promoters in the regulatory build of the Ensembl database (version 20161111), were classified as ‘promoters’. Other peaks were classified as putative enhancers, if they were overlapping with enhancers it the Ensembl regulatory build, or if they were overlapping with peaks of the enhancer mark H3K4me1 in forebrain P0 or adult cortex in published datasets from the Encode database (https://www.encodeproject.org/; bed files, datasets ENCFF172LKQ, ENCFF746YEV). As a quality control, the ATAC-Seq peaks were also overlapped with DNase-Seq peaks from E18.5 and adult brain (bed files, Encode datasets ENCFF591XUM, ENCFF865BUI). The corresponding bigwig files were downloaded from the Encode database for visualisations in the IGV viewer (v2.4.14).

To identify potential regulatory interactions, the original pipeline annotated each peak to the closest TSS. To include potential long-range interactions, distal peaks (i.e. peaks not classified as promoters) were annotated to additional genes, if the peaks were overlapping with regulatory regions for these genes in brain E14.5 or adult cortex described by Shen et al.^24^, or regulatory regions in adult cortex or cultured neural progenitors (data for confidence level *p* < 0.01) described by Ron et al.^23^. The conversion of mm9 genome coordinates to mm10 coordinates for these datasets was performed with the Ensembl Assembly Converter (https://www.ensembl.org/Homo_sapiens/Tools/ AssemblyConverter?db=core).

Normalisation of read counts and analysis of differential accessibility for the peak regions called on the merged dataset was performed using the Bioconductor “DESeq2” package. Minor peaks with a normalised accessibility <25 in all samples were removed for further analysis. Peaks with padj ≤ 0.05, abs(log2FC) ≥ 1 were considered as differentially accessible. Bigwig files of the merged replicates of each group or individual replicates were visualised in the IGV viewer, normalised by the fraction of reads in peaks (frip score, see Table S3), to adjust for differences in sequencing quality.

Count matrices for accessibility heatmaps were generated from bigwig files using the R packages ‘GenomicRanges’ (v1.36.1)^74^ and ‘rtracklayer’ (v1.44.4)^75^. The genomic coordinates of the selected peaks were imported from bed files, the bigwig files were imported with the function import(…,format = ‘BigWig’, as = ‘RleList’). From these data the counts at peak centers ±1  kb were binned in 100 bins of 20 bp using ‘CoverageHeatmap(…, coords = c(−1000, 1000), nbin = 100), normalised by the fraction of reads in peaks (frip score, see Supplementary Data 3), to adjust for differences in sequencing quality, and plotted using the ‘plotHeatmapList’ function.

De novo motif analysis in differentially accessible regions was performed on the respective bed files using the Homer findMotifsGenome.pl command (parameters: -size given -mask), with the mm10 mouse genome as background.

### ChIP-Seq re-analysis of published datasets

To identify likely transcription factor binding sites for *Lhx2*, *Fezf2* and *Rorb* in astrocytes, we re-analysed published high quality ChIP-Seq datasets for Lhx2 in olfactory neurons (GSE93570)^29^, Fezf2 in neurospheres (GSE46707)^30^, and RORg (*Rorc*) in Th17 cells, as a proxy for Rorb binding (SRP104092)^32^. To map peaks, we used the nf-core “chipseq” nextflow pipeline^72^ (version 1.2.1).

Putative regulatory elements likely binding the transcription factors in astrocytes were identified.

By overlapping the ChIP-Seq with open chromatin regions in astrocytes in vivo (ATAC-Seq data from the current study, regions annotated in Supplementary Data 4). Regions overlapping with ChIP peaks in both replicates of the relevant ChIP-Seq dataset were considered as transcription factor binding for the statistical analyses in Fig. 6/Supplementary Fig. 8.

### Metabolic profiling with GC-MS

1.5 × 10^6^ NSCs were plated on 10 cm dishes, differentiated to astrocytes and lentivirus-transduced as described above. At day 14 of differentiation the BMP4 medium was replaced for 6 h with ^13^C-U-glucose-BMP4-medium (BMP4-medium in which the basal DMEM/F12 medium was replaced by DMEM/F12 without glucose and glutamine (biowest L0091-500) + glutamax supplement (Thermo Fisher cat#35050061) + ^13^C-U-glucose (Cambridge Isotope Laboratories, CLM-1396-PK)).

Media was removed (with 5 µl reserved for analysis) and the cells washed twice with PBS, and once in saline. Cells were scraped off in 600 µl methanol/water (1:1 v/v, containing 5 nmol nor-leucine as internal standard) and transferred to a 1.5 mL tube (Eppendorf) containing 300 µl chloroform. The plate was washed with additional 600 µl methanol, which was added to the same tube. Both media and cell extract samples were stored at −80 °C. Thawed extracts were spun (13,200 × *g*, 4 °C, 5 min) to partition, and the upper phase (containing polar metabolites) removed for further processing.

Data acquisition was performed largely as previously described^76^, using an Agilent 7890A-5975C GC-MSD in EI mode after derivatization of dried extracts (or media) by addition of 20 μL methoxyamine hydrochloride (20 mg/mL in pyridine (both Sigma), RT, > 16 h) and 20 μL BSTFA + 1% TMCS (Sigma, RT, > 1 h). GC-MS parameters were as follows: carrier gas, helium; flow rate, 0.9 mL/min; column, DB-5MS (Agilent); inlet, 270 °C; temperature gradient, 70 °C (2 min), ramp to 295 °C (12.5 °C/min), ramp to 320 °C (25 °C/min, 3 min hold). Scan range was *m/z* 50–550. Data were acquired using MassHunter software (version 10, Agilent Technologies). Data analysis was performed using MANIC software (version 1.0), an in house-developed adaptation of the GAVIN package^77^. Metabolites were identified and quantified by comparison to authentic standards, and label incorporation estimated as the percentage of the metabolite pool containing one or more ^13^C atoms after correction for natural abundance.

Five independent differentiation experiments were performed at different days side-by-side for EGFP, Rorb and Fezf2 expression. Where indicated, relative changes in metabolite levels were determined for each condition by log2-transformation and normalisation to the mean of levels of the individual experiment, to account for inter-experimental variability. Note that although stable isotope-labelled glucose was used in the media, we do not report the results in this manuscript, since this neither added to, nor detracted from, our abundance data.

### Quantification of glutamine synthetase staining

For quantification of glutamine synthetase (GS) stainings (Fig. 5b), images of all compared samples were taken in one session with the same settings. Maximum intensity projections of each 2 random areas on 2 coverslips were analysed per independent sample. Intensity of GFP/V5 and GS were measured in regions of interest defined by the DAPI staining using FIJI. For each channel a common intensity threshold for all samples was defined using representative images.

### Statistical analyses

Statistical analysis of sequencing data was performed with standard methods of the used software tools as outlined in the relevant figures and methods sections. Other statistical analyses were performed using Graphpad Prism (v8.3.1).

### Reporting summary

Further information on research design is available in the Nature Research Reporting Summary linked to this article.

## Supplementary information

Supplementary information

Description of Additional Supplementary Files

Reporting Summary (updated)

Supplementary Software

Supplementary Movie:

Supplementary Movie 2

Supplementary Movie 3

Supplementary Dataset 1

Supplementary Dataset 2

Supplementary Dataset 3

Supplementary Dataset 4

Supplementary Dataset 5

Supplementary Dataset 6

Supplementary Dataset 7

Supplementary Dataset 8

Supplementary Dataset 9

## Data Availability

The data that support this study are available from the corresponding author upon reasonable request. Sequencing data generated in this study are deposited in the NCBI GEO repository with accession number GSE152223. Most of the data can also be explored in an interactive online resource (https://biologic.crick.ac.uk/astrocyte). Publicly available datasets used in the study include: L6_Astroependymal_cells.loom from http://loom.linnarssonlab.org (built on the raw data set SRP135960), E-MTAB-5514, GSE96539, GSE93570, GSE46707, SRP104092, ENCFF172LKQ, ENCFF746YEV, ENCFF591XUM, and ENCFF865BUI. Source data are provided with this paper.

## Source data

Source Data

## Code availability

The source code used for analyses in the manuscript is provided as Supplementary Software with the manuscript and is deposited on Github [https://github.com/FrancisCrickInstitute/Lattke_et_al_Astrocyte_Maturation].
