# Supplementary material for: Extensive transcriptional and chromatin changes underlie astrocyte maturation in vivo and in culture
Source: Nat Commun. 2021 Jul 15;12:4335. doi: 10.1038/s41467-021-24624-5 (PMC8282848; doi:10.1038/s41467-021-24624-5)
Supplement: Supplementary file 1 — Supplementary information [file 41467_2021_24624_MOESM1_ESM.docx]

**Extensive transcriptional and chromatin changes underlie astrocyte maturation *in vivo* and in culture (Lattke et al.) - Supplementary Information**

**Supplementary Information provided in this file:**

**Supplementary Fig. 1: Characterisation of cell clusters in striatal sc-RNA-Seq (related to Fig. 1)**

**Supplementary Fig. 2: Comparision of gene expression between astrocyte preparations from different brain regions (related to Fig. 2)**

**Supplementary Fig. 3: Validation of expression changes of selected immature and mature astrocyte-specific genes on protein level by immunolabelling (related to Fig. 2)**

**Supplementary Fig. 4: *In vivo* ATAC-Seq peak characterisation (related to Fig. 3)**

**Supplementary Fig. 5: Additional characterisation of chromatin accessibility changes and linked gene expression changes (related to Fig. 3)**

**Supplementary Fig. 6: Characterisation of astrocytes differentiated *in vitro* from cultured NSCs using BMP4 (related to Fig. 4)**

**Supplementary Fig. 7: Characterisation of transcriptional regulation by Rorb, Dbx2, Lhx2 and Fezf2 expression in cultured astrocytes (related to Fig. 5)**

**Supplementary Fig. 8: Additional characterisation of the genomic mechanisms underlying the role of Rorb, Dbx2, Lhx2 and Fezf2 in astrocyte maturation (related to Fig. 6)**

**Supplementary table 1: Primers used in this study (for the preparation of ATAC-Seq libraries)**


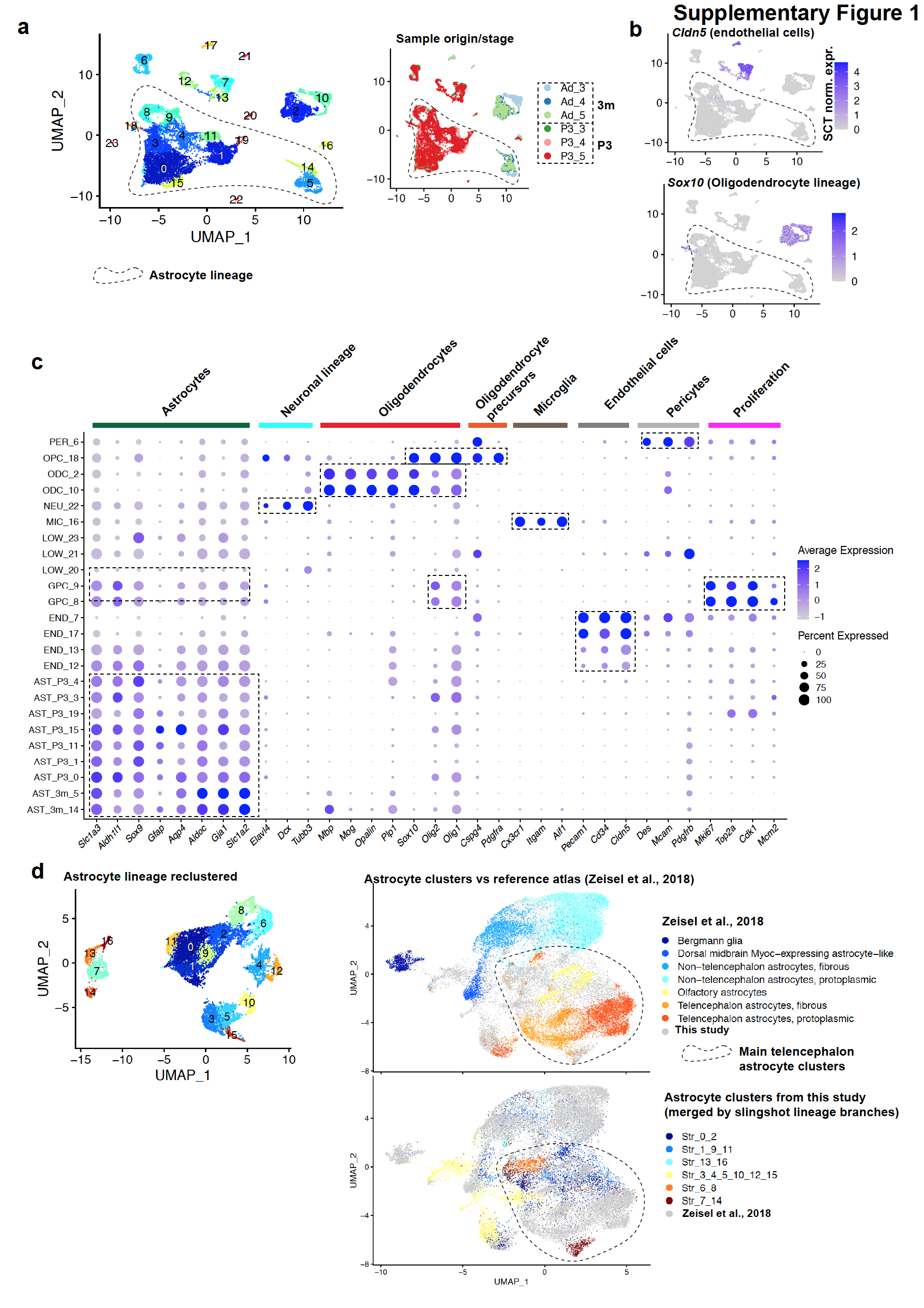


**Supplementary Fig. 1: Characterisation of cell clusters in striatal scRNA-Seq (related to Fig. 1)**

**(a)** UMAP dimension reduction plot of combined single cell transcriptomes from postnatal and adult striatal astrocyte preparations, with origin by sample projected on UMAP plot to assess batch effects

**(b)** Expression of *Cldn5* and *Sox10* projected on UMAP plot to distinguish endothelial cell and oligodendrocyte/oligodendrocyte precursor clusters from astrocyte clusters (*Sox9*+ *Cldn5*- *Sox10*-, see also Figure 1c)

**(c)** Expression of marker genes used to identify cell types in (a). Average expression (normalised with the Seurat SCTtransform algorithm) and fraction of cells with detectable transcripts for each cluster. Clusters are labelled with the assigned cell type and cluster number; AST_P3: early postnatal astrocytes (P3), AST_3m: adult astrocytes (age 3 months/3m), GPC: glial progenitor cells, OPC: oligodendrocyte precursor cells, MIC: microglia, PER: pericytes, END: endothelial cells, ODC: oligodendrocytes, NEU: neuronal lineage cells, LOW: low marker expression (unidentified).

(d) UMAP plots showing the astrocyte lineage cells after reclustering (left) and the location of the cells of these clusters in a combined UMAP plot with a reference dataset with juvenile astrocytes from different brain regions (right, from Zeisel et al., 2018, see Methods), to identify the regional identity of the astrocyte lineage cells

­
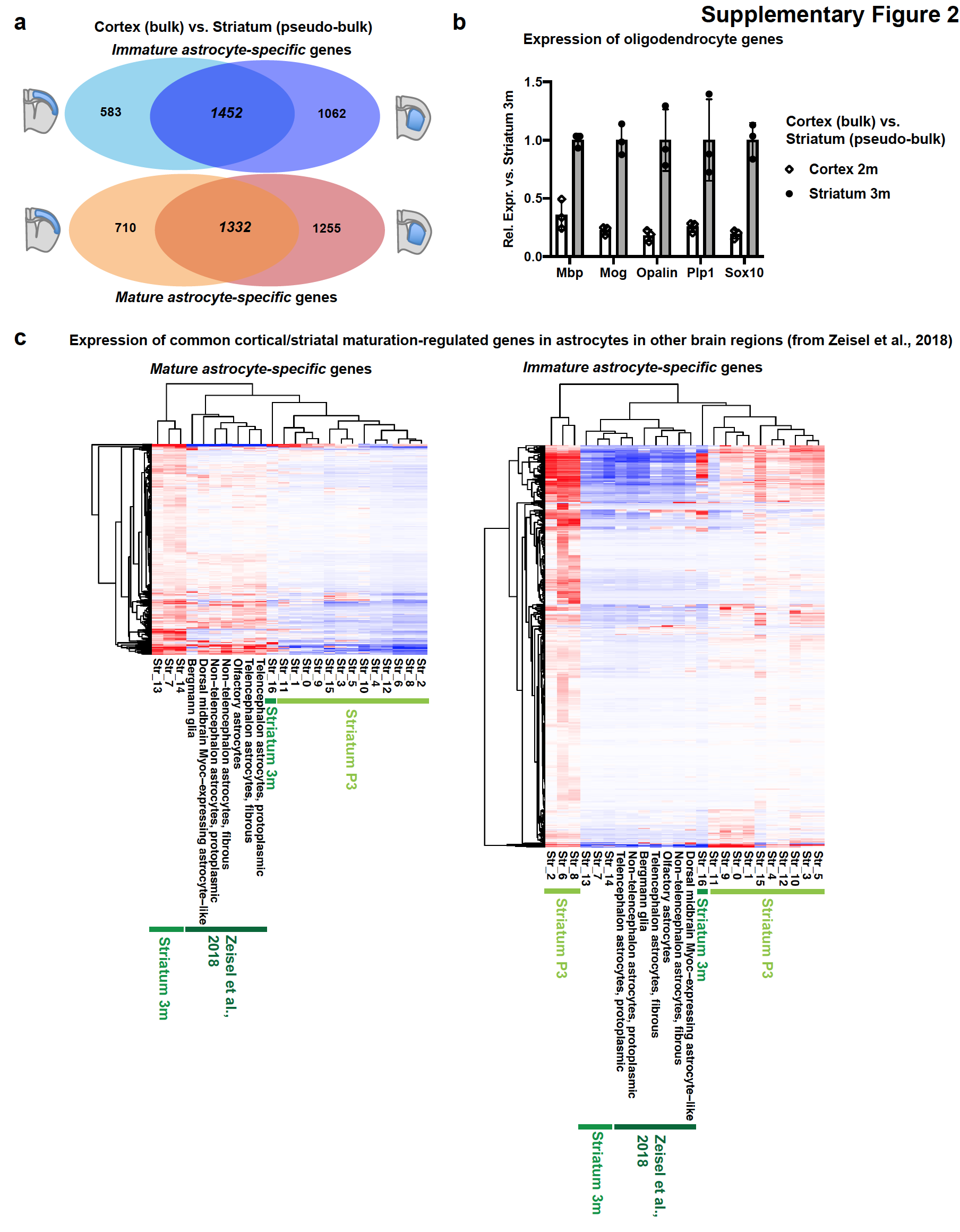


**Supplementary Fig. 2: Comparison of gene expression between astrocyte preparations from different brain regions (related to Fig. 1, 2)**

**(a)** Overlap of genes regulated between early postnatal and adult stages in cortical astrocyte preparations (bulk RNA-Seq) and scRNA-Seq data of striatal astrocyte preparations analysed as pseudo-bulk merged by sample, to compare both preparations in a identical analysis approach. DESeq2 analysis, each n=3; two-sided Wald test with Benjamini-Hochberg correction; significance threshold: adjusted p-value $\leq$ 0.05, absolute log2(fold change) $\geq$1;

**(b)** Expression of oligodendrocyte markers in cortical (bulk) vs striatal (pseudo-bulk) astrocyte preparations to estimate relative abundance of contaminating oligodendrocytes in the cortical preparations (mean +/- SD of each n=3 animals)

**(c)** Expression of the *immature* and *mature astrocyte-specific* gene sets from the comparison of cortical vs striatal astrocytes (Fig. 2b,c) in striatal astrocyte clusters vs juvenile astrocyte clusters from the reference dataset from Zeisel et al., 2018. Heatmaps show mean SCT-normalised, mean centred expression for each population (see Methods). Expression of the *immature/mature* genes in the juvenile astrocytes from all brain regions is more similar to adult striatal astrocytes than to early postnatal striatal astrocytes, based on hierarchical clustering of the cell populations. The adult striatal cluster 16 is more similar to early postnatal (immature) astrocytes.

**
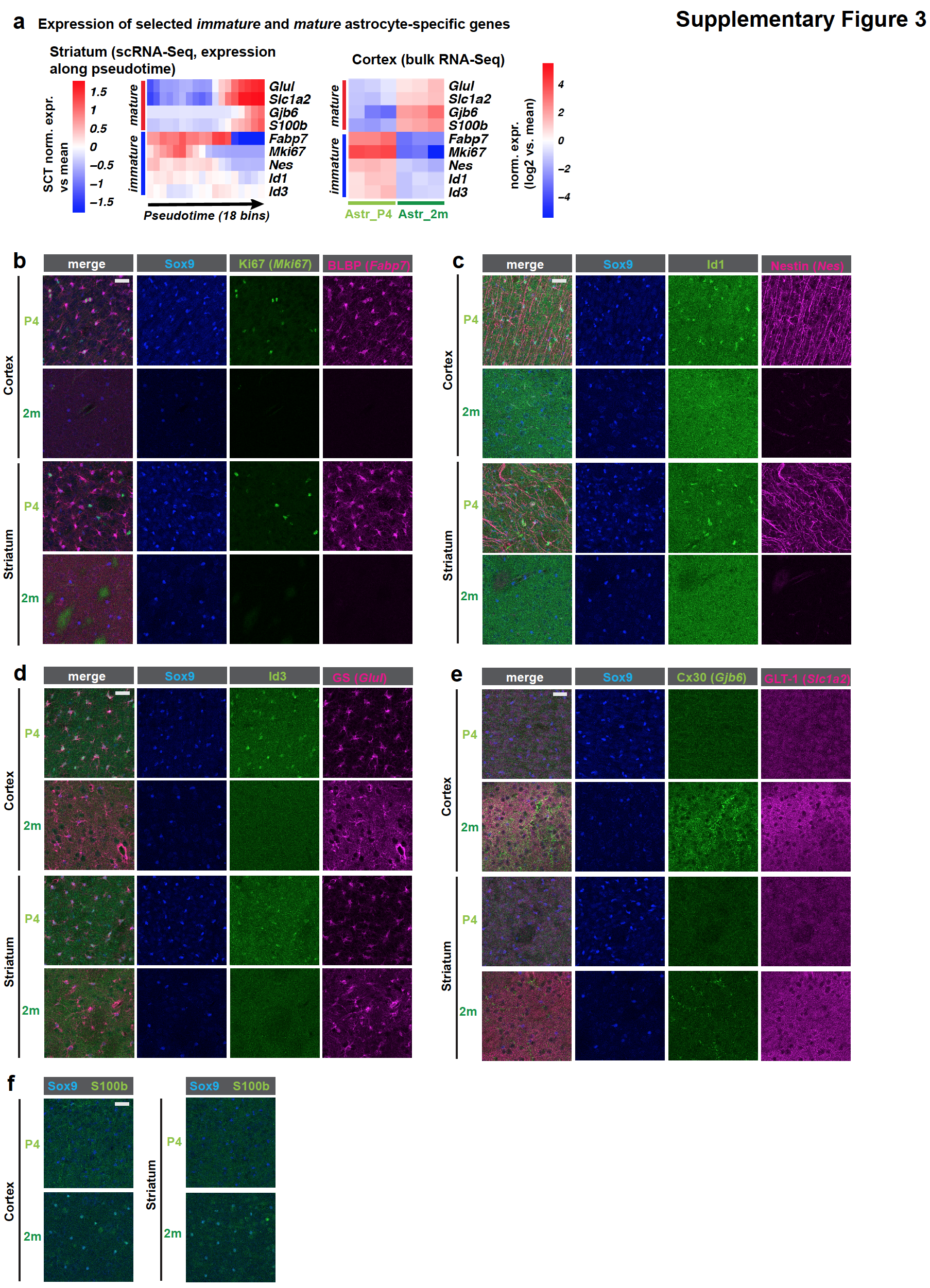
**

**Supplementary Fig. 3: Validation of expression changes of selected *immature* and *mature astrocyte-specific* genes on protein level by immunolabelling (related to Fig. 2)**

**(a)** Heatmaps showing the expression of maturation-regulated genes in striatal astrocytes along the pseudotime trajectory in Fig. 1e, f (mean expression in pseudotime bins), and in the bulk RNA-Seq analysis of cortical astrocytes (Fig. 2).

**(b, c)** Immunolabelling of cortical and striatal tissue at P4 and 2 months for the pan-astrocyte marker Sox9 and the protein products of the *immature* genes *Mki67*, *Fabp7*, *Id1* and *Nes* shown in (a)*.*

**(d)** Immunolabelling of cortical and striatal tissue at P4 and 2 months for the pan-astrocyte marker Sox9 and the protein products of the *immature* gene *Id3* and the *mature* gene *Glul* shown in (a)*.*

**(e, f)** Immunolabelling of cortical and striatal tissue at P4 and 2 months for the pan-astrocyte marker Sox9 and the protein products of the *mature* genes *Gjb6*, *Slc1a2* and *S100b* shown in (a)*.*

Presentation and replicability: (a) The heatmap for the scRNA-Seq analysis (left panel, data from Fig. 1) show the mean relative expression of each gene in all cells in each pseudotime bin (normalized, mean centred expression values); The heatmap for the cortical bulk RNA-Seq analysis (right panel, data from Fig. 2) shows log2-transformed mean centred, normalized expression values; (b-f) Representative images of 4 biological replicates per condition; Scale bars: 20 μm;


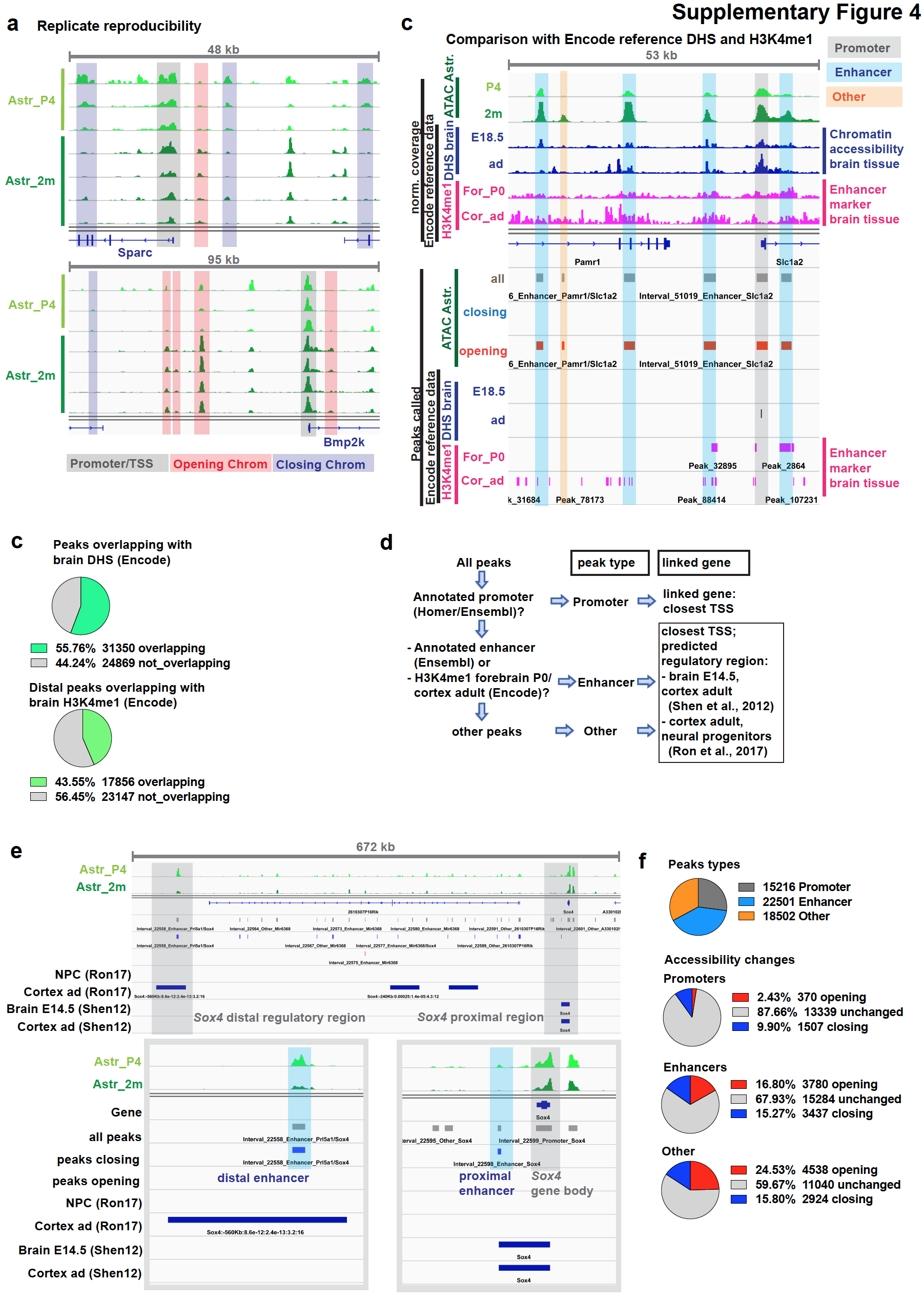
­­

**Supplementary Fig. 4: *In vivo* ATAC-Seq peak characterisation (related to Fig. 3)**

**(a)** Genome tracks show chromatin accessibility (normalised ATAC-Seq read count) in individual astrocyte samples from postnatal day 4 (Astr_P4) and 2 months of age (Astr_2m) at the *Sparc* and *Bmp2k* loci, to demonstrate reproducibility.

**(b, c)** Comparison of astrocyte ATAC-Seq data with reference datasets from the ENCODE database. DNase-Seq as alternative method to assess chromatin accessibility (whole brain samples from embryonic day (E) 18.5 and adult mice); ChIP-Seq for the H3K4me1 enhancer mark (tissue samples forebrain postnatal day (P) 0 and adult cortex); (b) Genome tracks of merged ATAC-Seq data and reference datasets, and corresponding peak regions called by the analysis pipeline (astrocyte samples) or published in the Encode database. (c) Quantification of the overlap of astrocyte ATAC peaks with the reference peak sets. The low signal-to-noise-ratio suggests that the H3K4me1 reference datasets are of relatively poor quality and that the figure of 44% overlap with ATAC peaks is an underestimate.

**(d, e)** Strategy for peak classification based on database annotations and overlap with the enhancer mark H3K4me1 from the reference datasets detailed above. Strategy for the identification of potential target genes of putative enhancers (distal ATAC peaks) located within published regulatory regions. (e) Genome Track of ATAC-Seq data and published regulatory regions around the locus of the *immature* gene *Sox4*, as illustration of this strategy: the highlighted peak in a distal *Sox4* regulatory region may represent a maturation-regulated *Sox4* enhancer that would not be linked to *Sox4* using only a conventional closest TSS approach.

**(f)** Classification of peaks identified in the merged astrocyte dataset based on the approach outlined in (d), and changes in their accessibility during cortical astrocyte maturation from P4 to 2 months of age.

Differential peaks: DESeq2 analysis, n=3 for Astr_P4, n=4 for Astr_2m; two-sided Wald test with Benjamini-Hochberg correction; significance threshold: adjusted p-value $\leq$ 0.05, absolute log2(fold change) $\geq$1;


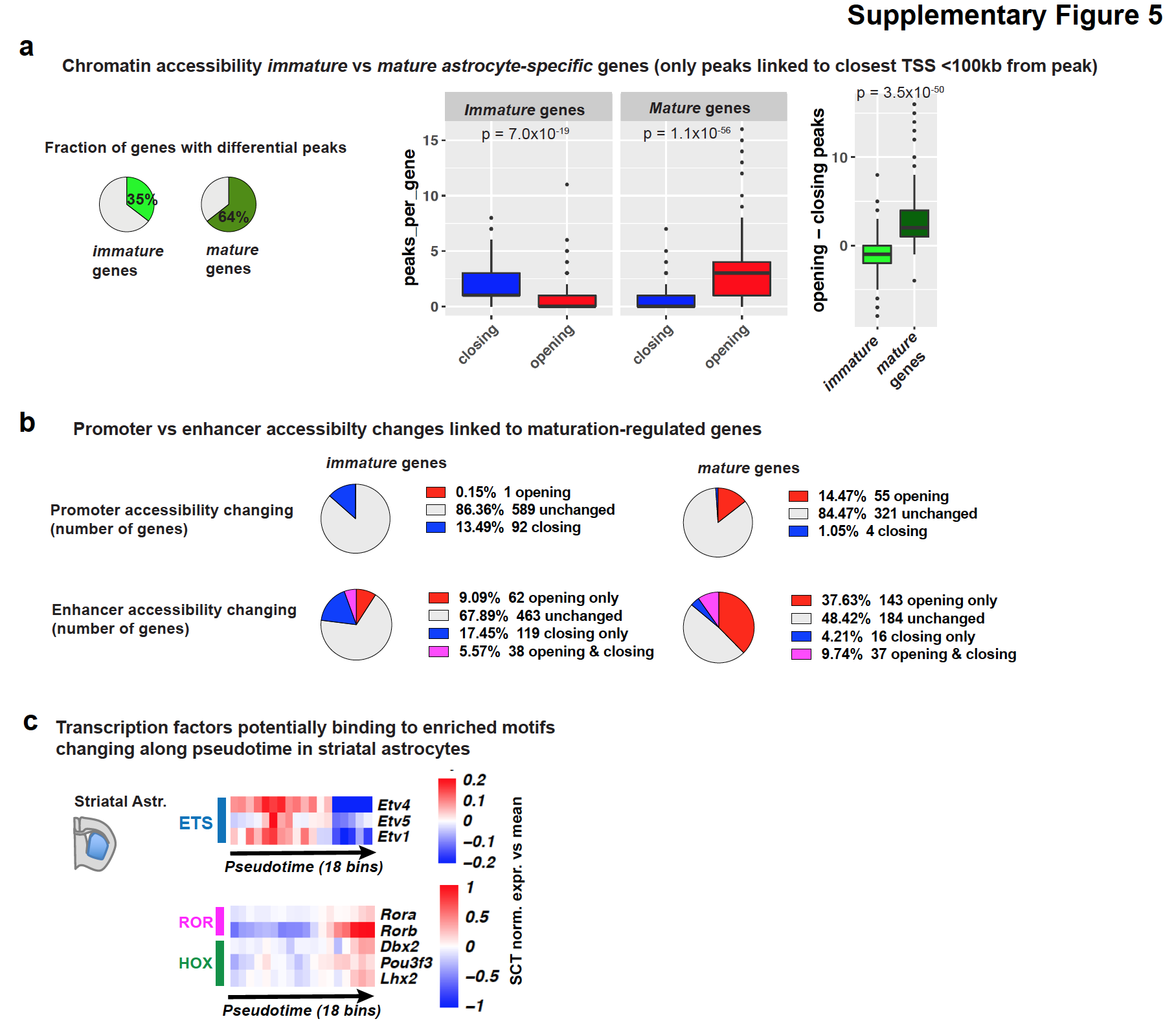


**Supplementary Fig. 5: *Additional characterisation of chromatin accessibility changes and linked gene expression changes (related to Fig. 3)***

**(a)** Genes regulated during astrocyte maturation (from Fig. 2c) are associated with chromatin regions that change in their accessibility between P4 and adult stages also if a more stringent selection of candidate regulatory elements is used (elements associated with closest transcriptional start site (TSS) with maximum distance of 100 kb).

**(b)** Separate analysis of chromatin accessibility at promoters and enhancers shows changes in promoter accessibility correlating with expression changes for a subset of maturation-regulated genes and changes in candidate enhancer accessibility for a larger subset of *immature* and *mature* genes.

**(c)** Expression of selected transcription factors with dynamic expression along pseudotime in striatal astrocytes that might bind to motifs in differentially accessible regions.

Statistical analysis and data presentation: (a) Two-sided Wilcoxon Rank Sum test (n= 341 immature vs 268 mature genes with associated differential peaks). Boxplots show: center line, median; box limits, upper and lower quartiles; whiskers, 1.5x interquartile range; points, outliers. (c) scRNA-Seq data (from Figure 1), heatmap of mean relative expression for pseudotime bins along lineage 6.

Differential peaks: DESeq2 analysis, n=3 for Astr_P4, n=4 for Astr_2m; two-sided Wald test with Benjamini-Hochberg correction; significance threshold: adjusted p-value $\leq$ 0.05, absolute log2(fold change) $\geq$1;


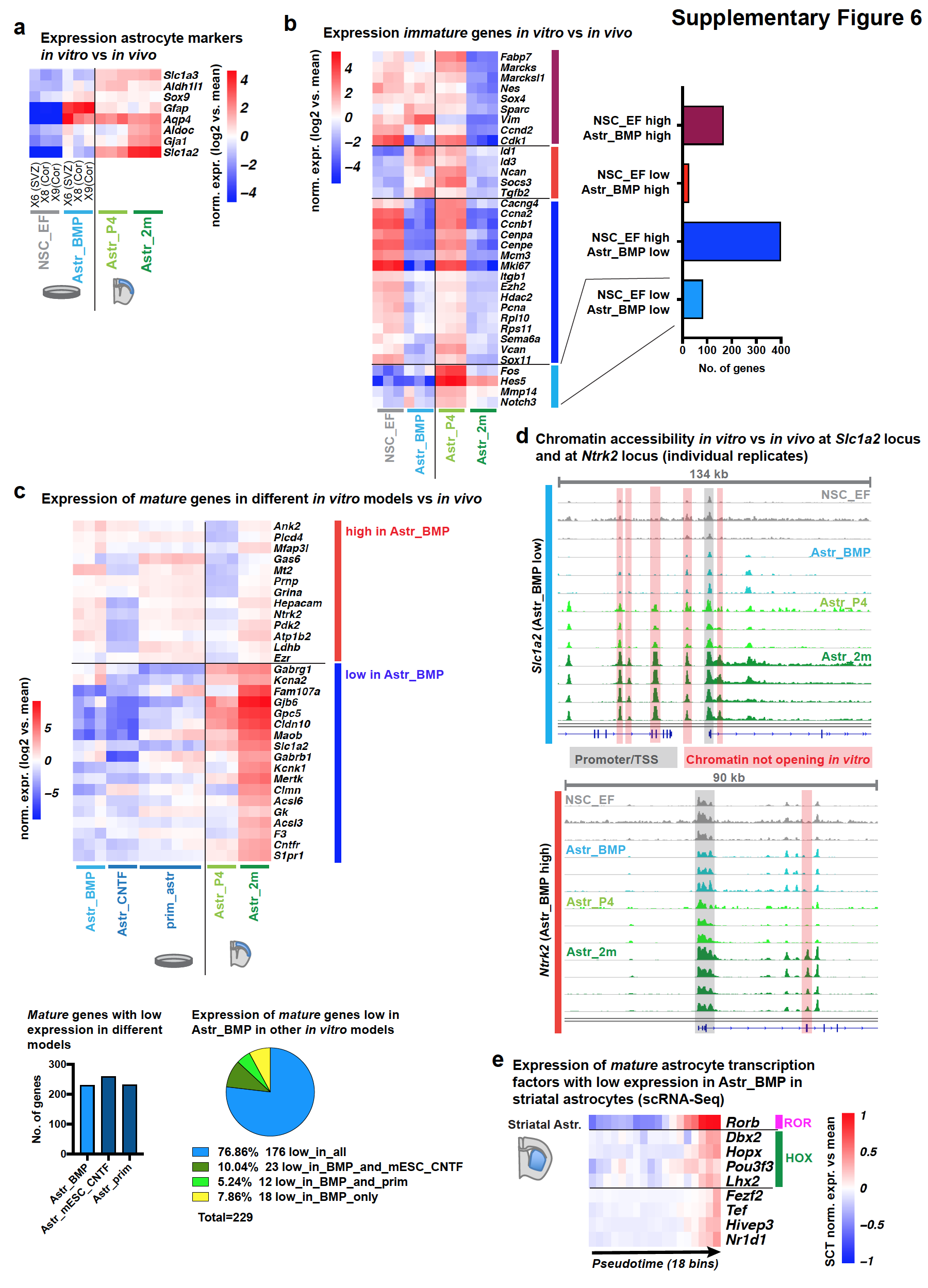


**Supplementary Fig. 6: Characterisation of astrocytes differentiated *in vitro* from cultured NSCs using BMP4 (related to Fig. 4)**

**(a)** Expression of astrocyte markers in astrocytes differentiated *in vitro* with BMP4 for 14 days (Astr_BMP), compared to the original NSCs cultured in EGF/FGF2 medium (NSC_EF), which had been derived from the adult subventricular zone (preparation X6 SVZ) or P3 cortex (preparations X8 Cor, X9 Cor). RNA-Seq, including cortical astrocytes *in vivo* from P4 or 2 months of age (Astr_P4 and Astr_2m, from Fig. 2).

**(b)** Expression of *immature astrocyte-specific* genes (from Fig. 2c) *in vitro* and *in vivo*. Heatmap of selected genes; and total number of genes, grouped by expression grouped by their expression patterns in cultured astrocytes; “NSC_EF/Astr_BMP high” refers to significantly higher expression compared to Astr_2m, “low” to equal or lower expression.

**(c)** Expression of *mature* genes (from Fig. 2c) in different *in vitro* astrocyte models. BMP4 differentiated astrocytes from this study (Astr_BMP), astrocytes differentiated from embryonic stem-cell-derived NSCs differentiated with CNTF (Astr_mESC_CNTF) and cultured primary astrocytes (prim_astr) from published datasets (Tiwari et al., 2018; Hasel et al., 2017). Heatmap of selected genes, and number of genes with low expression in the different *in vitro* models compared to adult cortical astrocytes.

**(d)** Chromatin accessibility at the Ntrk2 and Slc1a2 loci in vivo and in vitro in individual ATAC-Seq replicates.

**(e)** Expression of *mature* transcription factors with dynamic expression in striatal astrocytes, which show a low expression *in vitro* (compared to adult cortical astrocytes). scRNA-Seq data (from Fig. 1), heatmap of mean relative expression for pseudotime bins along lineage 6.

Statistical analysis and data presentation: Differential genes/peaks: DESeq2 analysis, each n=3 (n=4 for ATAC-Seq Astr_2m data); two-sided Wald test with Benjamini-Hochberg correction; significance threshold: adjusted p-value $\leq$ 0.05, absolute log2(fold change) $\geq$1; Heatmaps show log2-transformed mean centred, normalized expression values;


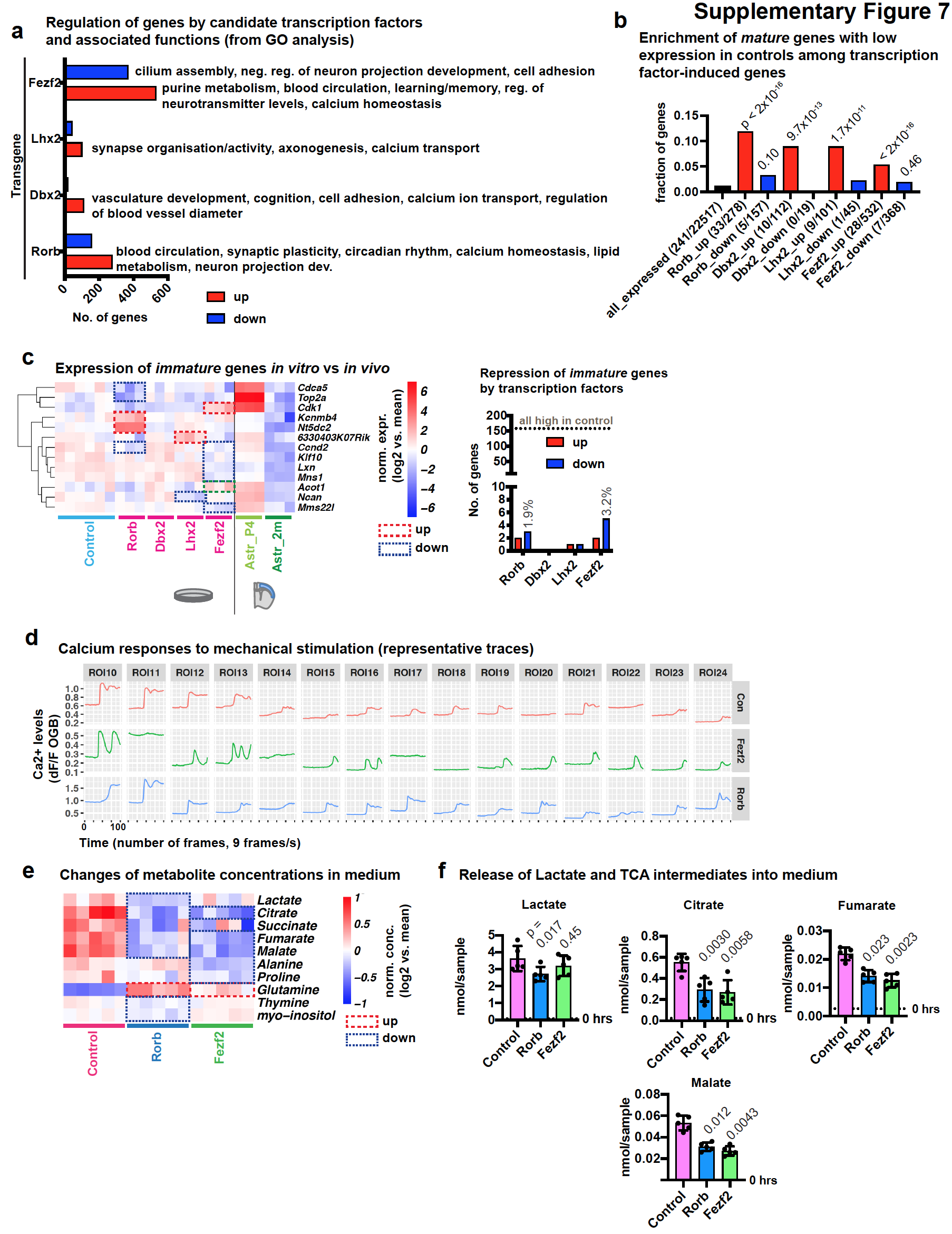


**Supplementary Fig. 7: Characterisation of transcriptional regulation by Rorb, Dbx2, Lhx2 and Fezf2 expression in cultured astrocytes (related to Fig. 5)**

**(a)** Total number of genes differentially expressed in astrocytes expressing candidate transcription factors compared to EGFP controls. Putative functions of these genes from GO analysis (details see Supplementary Data 7).

**(b)** Enrichment of maturation-regulated genes among genes regulated by expression of transcription factors *in vitro*.

**(c)** Expression of *immature astrocyte-specific* genes (from Fig. 2c) compared to in cortical astrocytes *in vivo* (from Figure 2). Heatmap of selected genes; barplot showing that of all immature genes with high expression in EGFP controls small subsets are repressed by the candidate transcription factors.

**(d)** Representative calcium response traces after mechanical stimulation for individual astrocytes in vitro expressing Rorb, Fezf2 or only the rtTA transgene as control (Con). Live cell imaging with the OGB calcium sensor dye.

**(e, f)** Metabolic changes induced by Rorb or Fezf2 in vitro, measured by GC/MS. Relative changes metabolite levels in the culture medium (e), and absolute levels in the culture medium before (0h baseline) and after exposure to the transgenic in vitro astrocytes for 6h (f).

Statistical analysis and data presentation: (b) Pairwise comparison of proportions with Benjamini-Hochberg; adjusted p values are shown. (c) Differential genes: DESeq2 analysis, each n=3 (n=6 for Control); two-sided Wald test with Benjamini-Hochberg correction; significance threshold: adjusted p-value $\leq$ 0.05, absolute log2(fold change) $\geq$1; Heatmaps show log2-transformed mean centred, normalized expression values; (e) Heatmap showing log2-normalised changes in metabolite levels relative to mean of each experiment (n = 5 independent experiments, Two-sided t-tests vs control with Benjamini-Hochberg correction for multiple testing; significant changes (padj < 0.05) are highlighted); (f) Absolute amount of glutamine detected in samples (means +/- SD shown, n = 5 independent experiments, one-way ANOVA with Tukey’s multiple comparisons test);


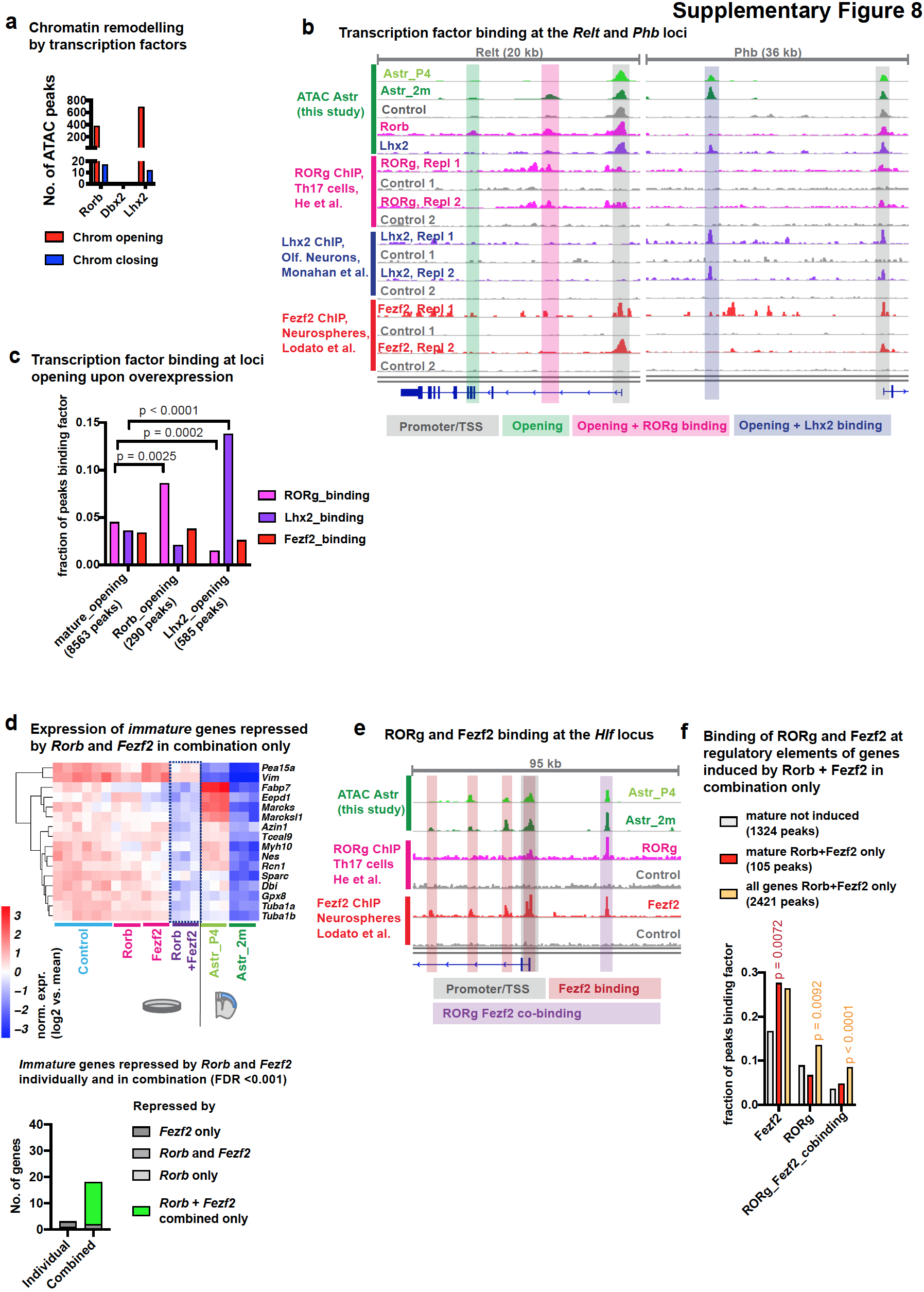


**Supplementary Fig. 8: Additional characterisation of the genomic mechanisms underlying the role of Rorb, Dbx2, Lhx2 and Fezf2 in astrocyte maturation (related to Fig. 6)**

**(a)** Number of ATAC-Seq peaks induced or suppressed by expression of transcription factors in cultured astrocytes.

**(b)** Binding of Lhx2, Fezf2 and the Rorb-related factor RORg in published ChIP-Seq datasets (each two replicates) at the *Relt* and *Phb* loci and chromatin accessibility *in vivo* and in *Rorb*- and *Lhx2*-expressing astrocytes *in vitro* (ATAC-Seq from this study)

**(c)** Enrichment of Lhx2, Fezf2 and RORg ChIP-Seq peaks in chromatin regions gaining accessibility during maturation in vivo or by expression of Rorb or Lhx2 in vitro.

**(d)** Regulation of *immature* genes by combined expression of *Rorb* and *Fezf2*. Samples from (b) were analysed together with astrocytes co-infected with *Rorb* and *Fezf2* viruses. RNA-Seq, including data from Fig. 2.

**(e)** Binding of Fezf2 and the Rorb-related factor RORg in published ChIP-Seq datasets, and chromatin accessibility in astrocytes *in vivo* in ATAC-Seq data from this study, at the *Hlf* locus.

**(f)** Enrichment of Fezf2 and the RORg ChIP-Seq peaks in published ChIP-Seq datasets in open chromatin (putative regulatory elements) linked to genes induced by Rorb and Fezf2 in combination only and in non-regulated *mature* genes.

Statistical analysis and data presentation: (a) Differential peaks: DESeq2 analysis, each n=3 (n=6 for Control); two-sided Wald test with Benjamini-Hochberg correction; significance threshold: adjusted p-value $\leq$ 0.05), absolute log2(fold change) $\geq$1;

(b, e) ATAC tracks represent merged reads of 3 replicates (4 for Astr_2m, 6 for Control), ChIP tracks represent individual replicates. (c, f) p values from two-sided Fisher-test of factor binding vs non-binding regions (in (f) vs regions linked to non-induced *mature* genes); (d) Differential genes: DESeq2 analysis, each n=3 (n=6 for Control); two-sided Wald test with Benjamini-Hochberg correction; significance threshold: adjusted p-value (FDR) $\leq$ 0.001), absolute log2(fold change) $\geq$1; Heatmaps show log2-transformed mean centred, normalized expression values;

**Supplementary table 1: Primers used in this study (for the preparation of ATAC-Seq libraries; Ad1: forward primer; Ad2.X: reverse primers with indicated barcodes for multiplexing)**

| **Primer name** | **Sequence** |
| --- | --- |
| Ad1_noMX: | AATGATACGGCGACCACCGAGATCTACACTCGTCGGCAGCGTCAGATGTG |
| Ad2.1_TAAGGCGA | CAAGCAGAAGACGGCATACGAGATTCGCCTTAGTCTCGTGGGCTCGGAGATGT |
| Ad2.2_CGTACTAG | CAAGCAGAAGACGGCATACGAGATCTAGTACGGTCTCGTGGGCTCGGAGATGT |
| Ad2.3_AGGCAGAA | CAAGCAGAAGACGGCATACGAGATTTCTGCCTGTCTCGTGGGCTCGGAGATGT |
| Ad2.4_TCCTGAGC | CAAGCAGAAGACGGCATACGAGATGCTCAGGAGTCTCGTGGGCTCGGAGATGT |
| Ad2.5_GGACTCCT | CAAGCAGAAGACGGCATACGAGATAGGAGTCCGTCTCGTGGGCTCGGAGATGT |
| Ad2.6_TAGGCATG | CAAGCAGAAGACGGCATACGAGATCATGCCTAGTCTCGTGGGCTCGGAGATGT |
