# Supplementary material for: Extensive transcriptional and chromatin changes underlie astrocyte maturation in vivo and in culture
Source: Nat Commun. 2021 Jul 15;12:4335. doi: 10.1038/s41467-021-24624-5 (PMC8282848; doi:10.1038/s41467-021-24624-5)
Supplement: Supplementary file 2 — Description of Additional Supplementary Files [file 41467_2021_24624_MOESM2_ESM.docx]

**Description of Additional Supplementary Files**

File name: **Supplementary Data 1**

Description:

Immature and mature striatal astrocyte genes identified by sc-RNA-Seq; sequencing and clustering metrics; pseudotime lineages; sets of dynamically expressed genes with similar expression patterns along pseudotime for the main striatal lineage 6; gene ontology terms enriched in immature and mature striatal astrocyte genes (related to Fig. 1).

File name: **Supplementary Data 2**

Description:

Genes differentially expressed between cortical astrocytes from P4 and 2-month-old mice identified by bulk RNA-Seq, including common *immature* and *mature* astrocyte genes differentially regulated in both striatal astrocyte sc-RNA-Seq and pseudobulk analysis and in cortical bulk RNA-Seq (related to Fig. 2).

File name: **Supplementary Data 3**

Description:

ATAC-Seq sequencing metrics for astrocytes *in vivo* and *in vitro*. (related to Fig. 3, 4, 6, 7).

File name: **Supplementary Data 4**

Description:

Annotations of ATAC-Seq peaks detected in combined P4 and 2-month-old cortical astrocytes. Putative types of regulatory elements and linked target genes; overlap with reference datasets (related to Fig. 3).

File name: **Supplementary Data 5**

Description:

*Immature* and *mature* genes potentially regulated by differential accessibility of regulatory regions; peak-gene pairs of differentially accessible ATAC peaks and linked *immature* and *mature* genes, with normalized chromatin accessibility and gene expression in cortical astrocytes P4 vs 2 months (related to Fig. 3).

File name: **Supplementary Data 6**

Description:

*In vivo* maturation-regulated genes, grouped by their expression in cultured NSCs and BMP4-differentiated astrocytes (related to Fig. 4).

File name: **Supplementary Data 7**

Description:

Genes regulated by Rorb, Dbx2, Lhx2 and Fezf2 expression in astrocytes *in vitro*, and associated gene ontology terms; including *mature* genes with low expression in EGFP controls and *immature* genes with high expression; (related to Fig. 5).

File name: **Supplementary Data 8**

Description:

Characterisation of the genomic mechanisms underlying the role of Rorb, Dbx2, Lhx2 and Fezf in astrocyte maturation; open chromatin regions in astrocytes and overlap with putative transcription factor binding sites from published ChIP-Seq datasets; numbers of binding sites in chromatin changing accessibility upon transcription factor expression; genes regulated by Rorb and Fezf2 in combination and transcription factor binding at regulatory elements of these genes (related to Fig. 6).

File name: **Supplementary Data 9**

Description:

Genes regulated by maturation signals (FGF2, 3D culture) in astrocytes *in vitro*, including signal-induced *mature* genes with low expression in controls (related to Fig. 7).

File name: **Supplementary Movie 1**

Description:

Calcium response (OGB fluorescence) of control in vitro astrocytes (expressing rtTA only) to mechanical stimulation (related to Fig. 5d)

File name: **Supplementary Movie 2**

Description:

Calcium response (OGB fluorescence) of Rorb expressing in vitro astrocytes to mechanical stimulation (related to Fig. 5d)

File name: **Supplementary Movie 3**

Description:

Calcium response (OGB fluorescence) of Fezf2 expressing in vitro astrocytes to mechanical stimulation (related to Fig. 5d)

File name: **Supplementary Software**

Description:

ZIP file with code used for the main analyses of the study, including detailed instructions and files required specific settings/parameters
