# Supplementary material for: Extensive transcriptional and chromatin changes underlie astrocyte maturation in vivo and in culture
Source: Nat Commun. 2021 Jul 15;12:4335. doi: 10.1038/s41467-021-24624-5 (PMC8282848; doi:10.1038/s41467-021-24624-5)
Supplement: Supplementary file 4 — Supplementary Software [file 41467_2021_24624_MOESM4_ESM.zip › github/Code_and_usage_overview.pdf]

Description of code used for analyses in "Extensive transcriptional and chromatin changes underlie astrocyte maturation in vivo and in culture" (Lattke et al., 2021)

Overview

The following analysis scripts and related files are described in this document:

- **Figure1_scRNA-Seq_striatum:** *This pipeline was used for the scRNA-Seq analysis of striatal astrocyte maturation (related to Figure 1).*
- **Figure2_RNA-Seq_cortex:** *This pipeline was used for the bulk-RNA-Seq analysis of cortical astrocyte maturation (related to Figure 2).*
- **Figure3_ATAC-Seq_in_vivo:** *This pipeline was used for the bulk-ATAC-Seq analysis of cortical astrocyte maturation (related to Figure 3).*
- **Figure4_ATAC-Seq_BMP_astr_vs_in_vivo**: *This pipeline was used for the bulk-ATAC-Seq analysis of astrocyte maturation in vitro vs in BMP astrocytes in vitro (related to Figure 4).*
- **Figure4_RNA-Seq_BMP_astr_vs_in_vivo:** *This pipeline was used for the bulk-RNA-Seq analysis of astrocyte maturation in vitro vs in BMP astrocytes in vitro (related to Figure 4).*
- **Figure5_metabolite_analysis:** *This pipeline was used for the metabolomic analysis of astrocytes differentiated in vitro overexpressing specific transcription factors (related to Figure 5).*
- **Figure5_RNA-Seq_transcription_factor_expression:** *This pipeline was used for the bulk-RNA-Seq analysis of astrocytes differentiated in vitro overexpressing specific transcription factors (related to Figure 5).*
- **Figure6_ATAC-Seq_transcription_factor_expression:** *This pipeline was used for the bulk-ATAC-Seq analysis of transcription factor expression in vitro vs astrocytes in vivo (related to Figure 6).*
- **Figure6_RNA-Seq_Rorb_Fezf2_co-expression:** *This pipeline was used for the bulk-RNA-Seq analysis of astrocytes differentiated in vitro overexpressing Rorb and Fezf2 in combination (related to Figure 5).*
- **Figure7_ATAC-Seq_maturation_signals:** *This pipeline was used for the bulk-ATAC-Seq analysis of different culture conditions in vitro vs astrocytes in vivo (related to Figure 7).*
- **Figure7_RNA-Seq_maturation_signals:** *This pipeline was used for the bulk-RNA-Seq analysis of different culture conditions in vitro vs astrocytes in vivo (related to Figure 7).*
- **FigureS2A_RNA-Seq_cortex_bulk_vs_Str_pseudobulk:** *This pipeline was used for the pseudo-bulk-RNA-Seq comparison of striatal and cortical astrocyte maturation (related to Figure S2A).*
- **FigureS6C_RNA-Seq_BMP_vs_published_in_vitro_astrocytes:** *This pipeline was used for the comparison of astrocyte maturation in vitro and in vivo with different published astrocyte in vitro models (related to Figure S6C).*
- **FigureS8_re-analysis_published_ChIP_vs_astrocyte_ATAC-Seq**: *This pipeline was used for the comparison of published ChIP-Seq datasets to ATAC- and RNA-Seq data from the transcription factor expression in vitro experiments (related to Figure S8).*

Table of contents

Overview 1

1. Figure1_scRNA-Seq_striatum 3
2. Figure2_RNA-Seq_cortex 5
3. Figure3_ATAC-Seq_in_vivo 6
4. Figure4_ATAC-Seq_BMP_astr_vs_in_vivo 8
5. Figure4_RNA-Seq_BMP_astr_vs_in_vivo 9
6. Figure5_metabolite_analysis 10
7. Figure5_RNA-Seq_transcription_factor_expression 11
8. Figure6_ATAC-Seq_transcription_factor_expression 12
9. Figure6_RNA-Seq_Rorb_Fezf2_co-expression 13
10. Figure7_ATAC-Seq_maturation_signals 14
11. Figure7_RNA-Seq_maturation_signals 15
12. FigureS2A_RNA-Seq_cortex_bulk_vs_Str_pseudobulk 16
13. FigureS6C_RNA-Seq_BMP_vs_published_in_vitro_astrocytes 17
14. FigureS8_re-analysis_published_ChIP_vs_astrocyte_ATAC-Seq 18

1. **Figure1_scRNA-Seq_striatum**

Script:
Figure1_scRNA-Seq_striatum.R

This pipeline was used for the scRNA-Seq analysis of striatal astrocyte maturation (related to Figure 1).

Input files required:

- ***10X-scRNA-Seq data mapped with cellranger***

In '00_input' folder (provided):
- **group_tab.csv:** *for each sample specification of sample name, path to cellranger output, experimental group*
- **Gene of interest table 21-02-19.csv:** *collection of various gene sets for analyses and plotting (e.g. transcription factors, cell type markers, …)*

The exact reproduction of the analysis also requires (provided or publicly available):
- In '00_input' folder:
  - **l6_r3_astroependymal_cells.loom:** *reference dataset from Zeisel et al., 2018 (from http://loom.linnarssonlab.org/) (for Part 5, Part 7)*
- **02_reg_genes_cortex.csv:** *set of genes differentially regulated during cortical astrocyte maturation from bulk-RNA-seq analysis (from* Figure2_RNA-Seq_cortex, *for Part 7)*
- In '01_output' folder (modified versions of files created by pipeline):
  - 01_cluster_assignment.csv: *manually assigned cluster names for complete dataset*
  - 02_clusters for reclustering.csv: *clusters used for reclustering*
  - 06_module_consolidation_table.csv: *gene modules generated by hierarchical clustering and corresponding names for manual module merging*
- Exact pseudotime coordinates used in the analysis, which are provided in: 'cell_meta_data_for_resource.csv'

Workflow description:

Part 1 (lines 1 - 237) used Seurat 3 for basic filtering and UMAP clustering and related visualisations for cell type identification.
Note that the 'resolution' parameter of the FindClusters() function is critical for the cluster assignment. Creates also a raw count matrix by cell and a pseudobulk matrix of all cells per sample merged.

The output file '01_cluster_assignment.csv' (provided) was modified to assign cluster names in Part 2.

Part 2 (lines 238 - 311): Cluster names were annotated and statistics about the cellular composition of the datasets was generated.

The output file '02_clusters for reclustering.csv' (provided) was modified to select cell clusters for further detailed analyses in Part 3 (other clusters were deleted).

Part 3 (lines 312 - 378): Cells from the selected clusters were isolated and reclustered using Seurat 3 UMAP clustering and selected visualisations were plotted.
Note that the 'resolution' parameter of the FindClusters() function is critical for the cluster assignment.

Part 4 (lines 379 - 499): Slingshot was used for pseudotime lineage analysis using cluster assignments after reclustering in Part 3 with cluster 6 as starting point.
The slingshot analysis was performed with the critical command parameters: slingshot(sce, reducedDim = 'PCA', clusterLabels = sce$seurat_clusters, start.clus = 6, approx_points = 100). A PCA plot for presentation was generated coloured by cluster assignment for each cell, also showing

cluster centres (median PC loadings for each cluster) and connections between clusters according to the Slingshot lineage analysis. Clusters for pseudotime analysis and the cluster colour scheme is defined in the beginning. Note that the precise Slingshot pseudotime values may not be completely reproducible between runs.

Part 5 (lines 500 - 643): Characterisation of the clusters by integration with the reference dataset form Zeisel et al., 2018.
The Seurat object imported from the relevant loom file was merged with the Seurat object from Part 4 using the Seurat 3 integration approach (with the IntegrateData() function). ***This integration step takes a significant amount of time to complete.*** *For the presentation of the UMAP plot of the merged dataset, clusters from the current dataset were manually color-coded according to the branch of the Slingshot lineage trajectory. The reference populations were color-coded using the "Description" metadata column, according to their clustering by brain region in the original study by Zeisel et al., in a separate presentation.*

Part 6 (lines 644 - 1157): Analysis of gene expression changes along pseudotime. tradeSeq was used to identify genes with dynamic expression along pseudotime, followed by binning of gene expression along pseudotime and supervised clustering to group genes with similar pseudotemporal expression patterns.
Lineage 6 from the Slingshot analysis from Part 4 was manually set to use for the pseudotemporal gene expression analysis. Initially, the PCA/lineage plot from Part 4 was replotted with lineage 6 highlighted and plotted color-coded by pseudotime along lineage 6. Next (lines 711 - 734), a negative binomial GAM model was fit for the SCT normalised expression of all genes to identify genes with pseudotemporally dynamic expression using the fitGAM() function, followed by the associationTest() function, to extract p-values for the pseudotemporal gene expression dynamics. ***For all genes, this step takes several hours to complete on a normal laptop****, therefore it might be reasonable to limit the analysis to a specific subset of genes, e.g. the most variable genes.*
In the following steps (lines 736 - 938), pseudotime bins are defined, a matrix with the expression changes per bin per gene is created (for each gene the mean SCT normalised expression per bin is calculated for each bin with more than 10 cells, and centered on the mean of all bins). Genes with minimal changes are removed and genes are initially ordered by hierarchical clustering according to their expression pattern. Three critical parameters are set for the grouping of genes according to their pseudotemporal expression pattern, which have to be determined empirically. The number of pseudotime bins is set in line 750 (here 20 bins), threshold to remove genes with only minimal changes is set in line 814 (minimal deviation from mean in any bin, here 0.2), and in line 832 the number of initial gene modules created by hierarchical clustering (here 50 modules). ***A heatmap ('06_heatmap_by_pseudotime_with_gene_modules.pdf') and a table are created ('06_module_consolidation_table.csv'), which can be used to merge these original gene modules to broader manually defined consolidated modules.*** *After the manual module consolidation (line 939 - 1157), gene number statistics are generated and plots for presentation are created (adjust plotted cell clusters, colors, plotted bins (here 2-19), and order of plotted modules in lines (1000-1012). Plots include a plot with the distribution of cells in each bin by cell cluster, and various heatmaps, of all genes by module and of selected gene sets.*

Part 7 (lines 1158-1394): Downstream analyses of genes identified by pseudotime analysis in Part 6. Gene Ontology analysis, comparison of regulated gene sets with gene sets identified in cortical astrocyte bulk RNA-Seq analysis (from 'Figure2_RNA-Seq_cortex'), and expression of shared genes between both datasets in comparison to the reference dataset from Zeisel et al. (from Part 5)
Gene ontology analysis of combined immature/mature gene modules (highest expression in early stages or at end of pseudotime trajectory). Modules are manually merged (lines 1172-1176) and combined gene sets analysed using the clusterprofiler package. Comparisons with the gene sets from the cortical astrocyte bulk analysis were manually defined (lines 1207 - 1238). Gene number statisitics and a table with the different gene sets were created (07_Cor vs Str immat mat genes_genes.csv), which includes the common immature/mature signature used throughout the paper. Heatmaps showing the expression of these gene sets and the hierarchical clustering of the different populations based on these genes are shown for the merged dataset including the reference populations from Zeisel et al. from Part 5.

Related figure panels and tables:

Generated from output files indicated (in folder 01_output)

Figures:
1C: 01_UMAP_by_cluster_and_sample.pdf, 01_UMAP_Sox9_Cldn5_Sox10.pdf
1D: 03_UMAP_by_cluster_sample_reclustered.pdf, 03_UMAP_reclustered_Ki67.pdf
1E: 06_slingshot analysis_start_clust_6_lineage_6_with pseudotime.pdf
1F: 06_stat_cluster_by_pseudotime_bin_2-19.pdf,
06_heatmap_pseudotime_gene_expr_cons_modules.pdf
S1A: 01_UMAP_by_cluster_and_sample.pdf
S1B: 01_UMAP_Sox9_Cldn5_Sox10.pdf
S1C: 02_Dotplot_cell markers_clusters_named.pdf
S1D: 05_UMAP_Str_astr_clusters_vs_Zeisel18.pdf
S2C: 07_heatmap_expr_vs_Zeisel18_mat_common.pdf,
07_heatmap_expr_vs_Zeisel18_immat_common.pdf
S3A: 06_heatmap_pseudotime_gene_expr_mat_immat_sel_IF.pdf
S5C: 06_heatmap_pseudotime_gene_expr_mat_immat_sel_ETS_TFs.pdf,
06_heatmap_pseudotime_gene_expr_mat_mat_sel_ROR_HOX_TFs.pdf
S6E: 06_heatmap_pseudotime_gene_expr_mat_mat_TF_sel_BMP_low.pdf

Table S1: 01_raw_merged_dataset_stats_by_sample.csv, 02_cell_fract_by_sample.csv, 02_cell_number_by_sample.csv, 04_slingshot lineages_clusters included.csv, 06_Stats_genes_by_module_cons.csv, 06_genes_by_module_cons.csv, 07_GO_immature.csv, 07_GO_mature.csv
Table S2: 07_Cor vs Str immat mat genes_genes.csv (only common maturation signature genes)

2. <u>Figure2_RNA-Seq_cortex</u>

Script:
Figure2_RNA-Seq_cortex.R

This pipeline was used for the bulk-RNA-Seq analysis of cortical astrocyte maturation (related to Figure 2).

Input files required:

In '00_input' folder (provided):
- **matrix.csv**: *count matrix (output from mapping pipeline crickbabs/BABS-RNASeq; https://github.com/crickbabs/BABS-RNASeq; git commit id: 335ce47db079d6cc2a7f82f4b762620c4f7f27e2)*
- **group_tab.csv:** *for each sample specification of sample name (from matrix.csv), experimental group*
- **comparisons.csv:** experimental groups to compare in DESeq2 analysis
- **Gene of interest table 21-02-19.csv:** *collection of various gene sets for analyses and plotting (e.g. transcription factors, cell type markers, …)*

The exact reproduction of the analysis also requires (provided or publicly available):
- **06_genes_by_module_cons.csv** (in '00_input' folder): *output from scRNA-Seq analysis (Figure1_scRNA-Seq_striatum); required to highlight maturation-regulated genes from this analysis in volcano plot (in Part3)*

Workflow description:

Part 1 (lines 1 - 160) used DESeq2 for differential gene expression analysis and initial filtering for expressed and differentially expressed genes.
Ensembl gene IDs are converted to MGI gene symbols using the biomaRt package. DESeq analysis is run comparing all groups defined in 'group_tab.csv'. Raw counts, normalised counts, log2-transformed normalised counts and the statistics output for the comparisons defined in comparisons.csv are collected in one table. Lowly expressed genes (normalised counts <10 in all samples) are removed and differentially expressed genes identified (padj < 0.05, abs(log2FC) > 1 in any of the defined comparisons).

Part 2 (lines 161 - 216): Manual definition of gene sets for further analyses (e.g. differentially expressed in specific comparison).
Saves table with genes in each gene set and table with related gene number statisitics

Part 3 (lines 217 - 360): Plotting of volcano plot and heatmaps for presentation
Volcano plot parameters are manually defined in lines 227 - 261. Manually defined gene sets are plotted from lines 350 onwards with the custom function plot_expr_heatmap().

Related figure panels and tables:

Generated from output files indicated (in folder 01_output)

Figures:
2C: 03_volcano plot with striatal and selected genes.pdf
3G: 03_heatmap_mature_immature_sel_ETS_TFs.pdf,
03_heatmap_mature_mature_sel_ROR_HOX_TFs.pdf
S3A: 03_heatmap_mature_immature_sel_IF.pdf

Table S2: 02_reg_genes_cortex.csv

3. Figure3_ATAC-Seq_in_vivo

Script:
Figure3_ATAC-Seq_in_vivo.R

This pipeline was used for the bulk-ATAC-Seq analysis of cortical astrocyte maturation (related to Figure 3).

Input files required:

Output from ATAC mapping pipeline (crickbabs/BABS-ATACSeqPE nextflow pipeline; https://github.com/ crickbabs/BABS-ATACSeqPE; git commit id: 22edccf72855d42e6692a27385cf50666c8f391c), in '00_input' folder:

- **merged_peaks.bed:** *bed file of peaks called of the merged dataset ("sample level" analysis)*
- **merged_peaks.homer.annotatePeaks.txt:** annotations from pipeline for called peaks
- **merged_peaks.results.txt:** *table including the raw counts for annotated peaks for each sample*

Note that the BABS-ATACSeqPE pipeline is now superseded; a newer version is available as part of the nf-core project: nf-core/atacseq pipeline (https://doi.org/10.5281/zenodo.263413272)

DEseq2 results table from related RNA-Seq analysis (see 'Figure2_RNA-Seq_cortex', in '00_input' folder*)*

- **01_Deseq_results_expressed.csv**

In ‘00_input’ folder (provided):

- **group_table.csv:** *for each sample specification of sample name (from ‘merged_peaks.results.txt’), experimental group*
- **comparisons.csv:** *experimental groups to compare in DESeq2 analysis*
- **group_tab_RNA.csv:** *group table from related RNA-Seq analysis (see ‘Figure2_RNA-Seq_cortex’)*
- **comparisons_RNA.csv:** *comparisons table from related RNA-Seq analysis (see ‘Figure2_RNA-Seq_cortex’)*

The exact reproduction of the analysis also requires (in ‘00_input’ folder):

- **mus_musculus.GRCm38.Regulatory_Build.regulatory_features.20161111.gff:** *the Ensembl genomic feature annotation database from https://www.ensembl.org*
- **Bed files from the Encode database (https://www.encodeproject.org) with the following identifiers:**
    - **ENCFF172LKQ:** *H3K4me1-ChIP-seq peaks for forebrain P0*
    - **ENCFF746YEV:** *H3K4me1-ChIP-seq peaks for adult cortex*
    - **ENCFF591XUM:** *DNase-seq peaks for brain E18.5*
    - **ENCFF865BUI:** *DNase-seq peaks for adult brain*
- **mCO.enh.1e-2_mm10.bed:** *regulatory regions in cortex tissue from Ron et al., 2017, liftover to mm10 genome coordinates*
- **pombo_NPC_Ncol.enh_1e-2_mm10.bed:** *regulatory regions in neural progenitors from Ron et al., 2017, liftover to mm10 genome coordinates*
- **Shen12_brainE14_mm10.bed:** *regulatory regions in E14 brain tissue from Shen et al., 2012, liftover to mm10 genome coordinates*
- **Shen12_cortex_mm10.bed:** *regulatory regions in cortex tissue from Shen et al., 2012, liftover to mm10 genome coordinates*
- **07_Cor vs Str immat mat genes_genes.csv:** *gene sets including the common immature and mature signature (output from ‘Figure1_scRNA-Seq_striatum’)*

Workflow description:

Part 1 (lines 1 - 248) Addition of peak annotations from reference datasets, classification of peaks and identification of putative target genes
Based on annotations from ‘merged_peaks.homer.annotatePeaks.txt’. Additional annotations are added from overlapping genomic regions from reference datasets specified in lines 42-106 using the annotate_set() function (lines 113-156). Peaks are classified into “Promoters” (annotated promoters/TSSs), “Enhancers” (annotated enhancers and peaks overlapping with H3K4me1 peaks in reference sets) and “Others”. For each peak, target genes are assigned according to the original annotation form the mapping pipeline (closest TSS), plus genes for which regulatory regions from reference datasets are overlapping with the peak. An additional more stringent target assignment is performed by using only the closest TSS with less than 100kb distance as target gene.

Part 2 (lines 249 - 433) Analysis of differential chromatin accessibility using DESeq2
Chromatin accessibility at the peaks identified by the mapping pipeline is quantified and differential accessibility between the selected groups is determined using DESeq2, based on read counts from the output of the mapping pipeline (merged_peaks.results.txt) and the defined groups (group_table.csv, comparisons.csv). Raw counts, normalised counts, log2-transformed normalised counts and the statistics output for the comparisons defined in comparisons.csv are collected in one table. Lowly accessible regions (normalised counts <25 in all samples) are removed and differentially accessible regions identified (padj < 0.05, abs(log2FC) > 1 in any of the defined comparisons). DESeq results tables similar to the RNA-Seq analyses (see ’Figure2_RNA-Seq_cortex’) are created, including the peak type and target annotations from Part 1, as well as bed files containing peak IDs and target genes in peak labels. Statistics for the numbers of different peak classes are generated.

Part 3 (lines 434 - 547) Integration of chromatin accessibility and gene expression data
Differential chromatin accessibility (from Part 2) and gene expression data (from ’Figure2_RNA-Seq_cortex’) are integrated based on the predicted peak - target gene pairs identified in Part 1, and

then filtered as described for the individual datasets. The merged dataset contains normalised counts for each sample for ATAC and RNA data, the relevant logFC and padj data for the defined comparisons, gene and peak IDs and peak classes (one line for each gene-peak-pair)

Part 4 (lines 548 - 758) Analysis of chromatin accessibility changes at peaks linked to immature/mature gene sets
Binary/qualitative code for differential accessibility and expression is added to combined results table from Part 3 (-1/0/1 for increased/unchanged/decreased accessibility or expression). Sets of gene - peak pairs are manually defined (lines 586 - 603). Statistics for the number of genes in each set are generated. Number of peaks with different regulation directions are quantified for each gene. Numbers of opening/closing peaks per gene are plotted for selected comparisons (selected in lines 645 and 704, for opening vs closing peaks and opening - closing peaks respectively), and relevant Wilcoxon tests are performed for statistical comparison.

Part 5 (lines 759 - 852) Analysis of chromatin accessibility changes at promoters vs enhancers linked to immature/mature genes separately
Manual definition of gene-peak sets and gene number statistics are generated as in Part 5, split by promoters and enhancers.

Part 6 (lines 853 - 1060) Analysis of chromatin accessibility changes at peaks linked to immature/mature gene sets (with stringent definition of target genes (100 kb cutoff)
As Part 4, with more stringently defined target genes (closest TSS with maximum of 100 kb cutoff)

Related figure panels and tables:

Generated from output files indicated (in folder 01_output)

Figures:
3D: 04_stats_immat mat genes with vs without diff peaks.csv, 04_peaks_per_gene_boxplot.pdf, 04_peaks_reg_net_opening_per_gene_boxplot.pdf, 04_peaks_reg_net_opening-closing_by_group_wilcoxon_p-val.csv, 04_peaks_reg_opening_vs_closing_by_group_wilcoxon_p-val.csv
S4C: 02_DHS_overlap.csv, 02_H3K4me1_overlap.csv
S4F: 02_peak_type_stats.csv, 02_stats_peaks opening closing by peak type.csv
S5A: 06_peaks_per_gene_boxplot_100kb cutoff.pdf, 06_peaks_reg_net_opening_per_gene_boxplot_100kb cutoff.pdf, 06_peaks_reg_net_opening-closing_by_group_wilcoxon_p-val_100kb cutoff.csv, 06_peaks_reg_opening_vs_closing_by_group_wilcoxon_p-val_100kb cutoff.csv, 06_stats_immat mat genes with vs without diff peaks_100kb_cutoff.csv
S5B: 05_stats_immat mat genes with vs without diff promoters vs enhancers.csv

Table S4: 01_ATAC_peaks_full_annot.csv
Table S5: 04_reg peaks linked to immat genes.csv, 04_reg peaks linked to mat genes.csv

4. Figure4_ATAC-Seq_BMP_astr_vs_in_vivo

Script:
Figure4_ATAC-Seq_BMP_astr_vs_in_vivo.R

This pipeline was used for the bulk-ATAC-Seq analysis of astrocyte maturation in vitro vs in BMP astrocytes in vitro (related to Figure 4). The first steps of the pipeline are adapted with minor modifications from ‘Figure3_ATAC-Seq_in_vivo’

Input files required:

See 'Figure3_ATAC-Seq_in_vivo'. The analysed RNA-Seq data are coming from pipeline 'Figure4_RNA-Seq_BMP_astr_vs_in_vivo'

Workflow description:

Parts 1 to 3 (lines 1 - 527): Peak annotation, differential accessibility analysis and integration with RNA-Seq data follow the workflow of 'Figure3_ATAC-Seq_in_vivo'

Part 4 (lines 528 - 672): Analysis of chromatin accessibility changes linked to immature/mature gene sets (adapted from 'Figure3_ATAC-Seq_in_vivo' Part 4)
Binary/qualitative code for differential accessibility and expression is added to combined results table from Part 3 (-1/0/1 for increased/unchanged/decreased accessibility or expression). Sets of gene - peak pairs are manually defined (lines 581 - 592). Statistics for the number of genes in each set are generated. Number of regions opening in mature astrocytes in vivo (Astr_P4 vs Astr_2m) with reduced accessibility in vitro (Astr_BMP vs Astr_2m) are quantified for each gene. Numbers of opening/closing peaks per gene are plotted for selected comparisons (opening - closing peaks), and relevant Wilcoxon tests are performed for statistical comparison.

Related figure panels and tables:

Generated from output files indicated (in folder 01_output)

Figures:
4E: 04_peaks_BMP_closed_mat_genes_BMP_low_vs_high_genes.pdf, 04_peaks_BMP_closed_mat_genes_BMP_low_vs_high_genes_wilcoxon_stat.txt

5. **Figure4_RNA-Seq_BMP_astr_vs_in_vivo**

Script:
Figure4_RNA-Seq_BMP_astr_vs_in_vivo.R

This pipeline was used for the bulk-RNA-Seq analysis of astrocyte maturation in vitro vs in BMP astrocytes in vitro (related to Figure 4). The first steps of the pipeline are adapted with minor modifications from 'Figure2_RNA-Seq_cortex'

Input files required:

count matrices (in '00_input'): **matrix_in vitro in vivo_old X6 data.csv**, **matrix_in vitro X6 resequenced.csv**
Output from mapping pipeline crickbabs/BABS-RNASeq; https://github.com/crickbabs/BABS-RNASeq; git commit id: 335ce47db079d6cc2a7f82f4b762620c4f7f27e2; The original analysis included in vitro libraries for replicate X6 which were prepared with a different kit. New libraries were prepared for these samples and sequenced, replacing the old data for these samples.

In '00_input' folder (provided):
- **group_tab.csv:** *for each sample specification of sample name (from matrix.csv), experimental group*
- **comparisons.csv:** experimental groups to compare in DESeq2 analysis
- **Gene of interest table 21-02-19.csv:** *collection of various gene sets for analyses and plotting (e.g. transcription factors, cell type markers, …)*

The exact reproduction of the analysis also requires (provided or publicly available):
- **07_Cor vs Str immat mat genes_genes.csv:** *gene sets including the common immature and mature signature (output from 'Figure1_scRNA-Seq_striatum')*

Workflow description:

Part 1 (lines 1 - 169): DESeq analysis as in 'Figure2_RNA-Seq_cortex'
The count matrix for the DESeq analysis was generated from the two input matrices in lines 34-50

Part 2 (lines 170 - 351): Analysis of expression changes of immature/mature astrocyte gene sets in vitro vs in vivo
Binary/qualitative code for differential expression is added to the results table from Part 1 (-1/0/1 for increased/unchanged/decreased expression). Selected gene sets are manually defined (lines 201 - 237). Statistics for the gene numbers per gene set are generated, and heatmaps for the expression of selected gene sets are plotted (lines 336 - 351).

Related figure panels and tables:

Generated from output files indicated (in folder 01_output)

Figures:
4C: 03_heatmap_immature_sel_BMP_reg.pdf, 02_genes_in_modules_stats.csv
4G: 03_heatmap_mature_TFs_sel.pdf
S6A: 03_heatmap_mature_Astr_markers.pdf
S6B: 03_heatmap_immature_sel_BMP_reg.pdf, 02_genes_in_modules_stats.csv

Table S6: 02_genes_in_modules.csv

6. Figure5_metabolite_analysis

Script:
Figure5_metabolite_analysis.R

This pipeline was used for the metabolomic analysis of astrocytes differentiated in vitro overexpressing specific transcription factors (related to Figure 5).

Input files required (provided):

- **metabolite_classes.csv:** *Table with classification of metabolites by pathway*
- **metabolites_cells_and_media.csv:** *Table with analysed metabolites, experiment/replicate identifiers, experimental condition, metabolite levels in cell extract/media samples/baseline medium sample, %C13 labelling fraction in cell extract/media sample*

Workflow description:

Metabolite levels in cell/media samples were normalised to the mean of all samples in the individual experiment to account for inter-experiment variation and log2 transformed (log2(X+0.001 nmol) and centred on the mean of all samples in each experiment. For media samples also the net change in levels compared to the baseline media sample (not exposed to cells). Metabolites are grouped by their pathway assignment in 'metabolite_classes.csv'. The variables to be further analysed and plotted are defined in lines 57-58. Statistical analysis of each metabolite level (log2 transformed, mean centered) in experimental conditions vs the EGFP control condition is performed by multiple t-tests with Benjamini-Hochberg correction (lines 61-99). Individual metabolite levels (raw and normalised) and C13-labelling fractions were plotted as points in individual graphs for each metabolite (lines 100-131). Matrices with individual variables for all samples and metabolites were created and plotted as heatmaps (lines 132 - 279).

Related figure panels and tables:

Generated from output files indicated (in folder /out)

Figures:
5E: heatmap_conc_log_norm_cells_only_sign.pdf, stat_conc_log_norm_cells.csv
S7E: heatmap_conc_log_norm_media_only_sign.pdf, stat_conc_log_norm_media.csv

Table S7: table_conc_cells_values_and_stats.csv, table_conc_log_norm_cells_values_and_stats.csv, table_conc_log_norm_media_values_and_stats.csv, table_conc_media_values_and_stats.csv

7. Figure5_RNA-Seq_transcription_factor_expression

Script:
Figure5_RNA-Seq_transcription_factor_expression.R

This pipeline was used for the bulk-RNA-Seq analysis of astrocytes differentiated in vitro overexpressing specific transcription factors (related to Figure 5). The first steps of the pipeline are adapted with minor modifications from 'Figure2_RNA-Seq_cortex'

Input files required:

count matrices (in '00_input'): **matrix_in vitro in vivo_old X6 data.csv**, **matrix_TF_OE.csv**
Output from mapping pipeline crickbabs/BABS-RNASeq; https://github.com/crickbabs/BABS-RNASeq; git commit id: 335ce47db079d6cc2a7f82f4b762620c4f7f27e2; Raw count matrices from transcription factor expression in vitro experiment and in vivo cortical astrocyte data as reference

In '00_input' folder (provided):

- **group_tab.csv:** *for each sample specification of sample name (from matrix.csv), experimental group*
- **comparisons.csv:** experimental groups to compare in DESeq2 analysis
- **Gene of interest table 21-02-19.csv:** *collection of various gene sets for analyses and plotting (e.g. transcription factors, cell type markers, …)*

The exact reproduction of the analysis also requires (provided or publicly available):

- **07_Cor vs Str immat mat genes_genes.csv:** *gene sets including the common immature and mature signature (output from 'Figure1_scRNA-Seq_striatum')*

Workflow description:

Part 1 (lines 1 - 169): DESeq analysis as in 'Figure2_RNA-Seq_cortex'
The count matrix for the DESeq analysis was generated from the two input matrices in lines 36-50

Part 2 (lines 170 - 388): Analysis of expression changes of immature/mature astrocyte gene sets in transcription factor expressing astrocytes in vitro vs in vivo
Binary/qualitative code for differential expression is added to the results table from Part 1 (-1/0/1 for increased/unchanged/decreased expression). Selected gene sets are manually defined (lines 199 - 232). Statistics for the gene numbers per gene set are generated, and pairwise proportion tests (function pairwise.prop.test()) were used to analyse overlap of all regulated genes with mature/immature gene sets. Heatmaps for the expression of selected gene sets are plotted (lines 338 - 352). Gene Ontology analysis is performed on selected gene sets (defined in lines 362 - 364)

Related figure panels and tables:

Generated from output files indicated (in folder 01_output)

Figures:
5C: 02_heatmap_mature_low_in_EGFP_reg_by_any_TF_selected.pdf, 02_genes_in_modules_stats.csv
S7A: 02_genes_in_modules_stats.csv, 02_GO_Dbx2_up.csv, 02_GO_Fezf2_down.csv, 02_GO_Fezf2_up.csv, 02_GO_Lhx2_down.csv, 02_GO_Lhx2_up.csv, 02_GO_Rorb_down.csv, 02_GO_Rorb_up.csv
S7B: 02_genes_in_modules_stats.csv, 02_genes reg in_mat_low vs all expressed_pairwise comp stat.txt
S7C: 02_heatmap_immature_high_in_EGFP_reg_by_any_TF.pdf, 02_genes_in_modules_stats.csv

Table S7: 02_genes_in_modules.csv, 02_GO_Dbx2_up.csv, 02_GO_Fezf2_down.csv, 02_GO_Fezf2_up.csv, 02_GO_Lhx2_down.csv, 02_GO_Lhx2_up.csv, 02_GO_Rorb_down.csv, 02_GO_Rorb_up.csv

8. Figure6_ATAC-Seq_transcription_factor_expression

Script:
Figure6_ATAC-Seq_transcription_factor_expression.R

This pipeline was used for the bulk-ATAC-Seq analysis of transcription factor expression in vitro vs astrocytes in vivo (related to Figure 6). The first steps of the pipeline are adapted with minor modifications from 'Figure3_ATAC-Seq_in_vivo'

Input files required:

Output from ATAC mapping pipeline (crickbabs/BABS-ATACSeqPE nextflow pipeline; https://github.com/ crickbabs/BABS-ATACSeqPE; git commit id: 22edccf72855d42e6692a27385cf50666c8f391c), in '00_input' folder:

- **merged_peaks.bed:** *bed file of peaks called of the merged dataset ("sample level" analysis)*
- **merged_peaks.homer.annotatePeaks.txt:** annotations from pipeline for called peaks
- **merged_peaks.results.txt:** *table including the raw counts for annotated peaks for each sample*
- **bigwig files for each experimental group merged** *("sample level" analysis)*

Note that the BABS-ATACSeqPE pipeline is now superseded; a newer version is available as part of the nf-core project: nf-core/atacseq pipeline (https://doi.org/10.5281/zenodo.263413272)

In '00_input' folder (provided):

- **group_table.csv:** *for each sample specification of sample name (from 'merged_peaks.results.txt'), experimental group*
- **comparisons.csv:** *experimental groups to compare in DESeq2 analysis*
- **group_table_bigwigs.csv***: groups, path to bigwig file directory, name of bigwig files, frip-score (fraction of reads in peaks) for each bigwig (from ATAC-SeqPE pipeline QC)*

The exact reproduction of the analysis also requires (in '00_input' folder, see 'Figure3_ATAC-Seq_in_vivo'):

- **mus_musculus.GRCm38.Regulatory_Build.regulatory_features.20161111.gff:** *the Ensembl genomic feature annotation database from https://www.ensembl.org*
- **Bed files from the Encode database (https://www.encodeproject.org) with the following identifiers:**
    - **ENCFF172LKQ:** *H3K4me1-ChIP-seq peaks for forebrain P0*

- **ENCFF746YEV:** *H3K4me1-ChIP-seq peaks for adult cortex*
  - **ENCFF591XUM:** *DNase-seq peaks for brain E18.5*
  - **ENCFF865BUI:** *DNase-seq peaks for adult brain*
- **mCO.enh.1e-2_mm10.bed:** *regulatory regions in cortex tissue from Ron et al., 2017, liftover to mm10 genome coordinates*
- **pombo_NPC_Ncol.enh_1e-2_mm10.bed:** *regulatory regions in neural progenitors from Ron et al., 2017, liftover to mm10 genome coordinates*
- **Shen12_brainE14_mm10.bed:** *regulatory regions in E14 brain tissue from Shen et al., 2012, liftover to mm10 genome coordinates*
- **Shen12_cortex_mm10.bed:** *regulatory regions in cortex tissue from Shen et al., 2012, liftover to mm10 genome coordinates*

Workflow description:

Parts 1 to 2 (lines 1 - 398): Peak annotation and differential accessibility analysis follow the workflow of 'Figure3_ATAC-Seq_in_vivo'

Part 3 (lines 399 -): Analysis of chromatin accessibility changes induced by transcription factor expression
Binary/qualitative code for differential accessibility and expression is added to combined results table from Part 3 (-1/0/1 for increased/unchanged/decreased accessibility or expression). Peak sets are manually defined (lines 439-463). Statistics for the number of peaks in each set are generated. Coverage matrices (normalised by frip score) are generated (lines 498-563) for selected comparisons defined in line 514 (for peak centre +/- 1kb). The peaks (rows) in the coverage matrices are ordered by the sum of normalised counts in the analysed window in the reference group defined in line 578 and the log2-transformed matrices for each group are plotted in the order defined in 'group_table_bigwigs.csv' (lines 566 - 606)

Related figure panels and tables:

Generated from output files indicated (in folder 01_output)

Figures:
6B: 03_coverage_heatmaps_Lhx2_opening.pdf, 03_coverage_heatmaps_Rorb_opening.pdf
S8A: 03_peak_module_stats.csv

9. <u>Figure6_RNA-Seq_Rorb_Fezf2_co-expression</u>

Script:
Figure6_RNA-Seq_Rorb_Fezf2_co-expression.R

This pipeline was used for the bulk-RNA-Seq analysis of astrocytes differentiated in vitro overexpressing Rorb and Fezf2 in combination (related to Figure 5).

Input files required:

count matrices (in '00_input')**: matrix_TF_OE_comb.csv, matrix_in_vitro_in_vivo.csv** (also used in 'Figure3_ATAC-Seq_in_vivo'), **matrix_TF_OE_indiv.csv** (also used in 'Figure5_RNA-Seq_transcription_factor_expression')
Output from mapping pipeline crickbabs/BABS-RNASeq; https://github.com/crickbabs/BABS-RNASeq; git commit id: 335ce47db079d6cc2a7f82f4b762620c4f7f27e2; Raw count matrices from transcription factor expression in vitro experiment and in vivo cortical astrocyte data as reference

In ‘00_input’ folder (provided):

- **group_tab.csv:** *for each sample specification of sample name (from matrix.csv), experimental group*
- **comparisons.csv:** experimental groups to compare in DESeq2 analysis
- **Gene of interest table 21-02-19.csv:** *collection of various gene sets for analyses and plotting (e.g. transcription factors, cell type markers, …)*

The exact reproduction of the analysis also requires:

- **07_Cor vs Str immat mat genes_genes.csv:** *gene sets including the common immature and mature signature (output from ‘Figure1_scRNA-Seq_striatum’)*

Workflow description:

Part 1 (lines 1 - 169): DESeq analysis as in ‘Figure2_RNA-Seq_cortex’
The count matrix for the DESeq analysis was generated from the three input matrices in lines 29-51

Part 2 (lines 170 - 342): Analysis of expression changes of immature/mature astrocyte gene sets in transcription factor expressing astrocytes in vitro vs in vivo
Binary/qualitative code for differential expression is added to the results table from Part 1 (-1/0/1 for increased/unchanged/decreased expression; cutoff here is padj<0.001). Selected gene sets are manually defined (lines 176 - 245). Gene number per gene set statistics, and a table with genes in each set, are generated. Heatmaps for the expression of selected gene sets are plotted (lines 335 - 342).

Related figure panels and tables:

Generated from output files indicated (in folder 01_output)

Figures:
6D: 02_heatmap_mature_low_in_EGFP_comb_RF_not_R_F_up.pdf, 02_genes_in_modules_stats.csv
S8D: 02_heatmap_immature_high_in_EGFP_comb_RF_not_R_F_down.pdf, 02_genes_in_modules_stats.csv

Table S8: 02_genes_in_modules.csv

10. Figure7_ATAC-Seq_maturation_signals

Script:
Figure7_ATAC-Seq_maturation_signals.R

This pipeline was used for the bulk-ATAC-Seq analysis of different culture conditions in vitro vs astrocytes in vivo (related to Figure 7). The pipeline is adapted with minor modifications from ‘Figure6_ATAC-Seq_transcription_factor_expression’

Input files required:

See ‘Figure6_ATAC-Seq_transcription_factor_expression’

Workflow description:

Parts 1 to 2 (lines 1 - 398): Peak annotation and differential accessibility analysis follow the workflow of ‘Figure3_ATAC-Seq_in_vivo’

Part 3 (lines 399 -): Analysis of chromatin accessibility changes induced by transcription factor expression
See 'Figure6_ATAC-Seq_transcription_factor_expression'. Peak sets are manually defined (lines 418-458). Coverage matrices are generated for selected comparisons defined in line 508. The peaks (rows) in the coverage matrices are ordered using the reference group defined in line 572 and then plotted.

Related figure panels and tables:

Generated from output files indicated (in folder 01_output)

Figures:
7E: 03_coverage_heatmaps_mature_opening_low_in_bas_2D.pdf

11. <u>Figure7_RNA-Seq_maturation_signals</u>

Script:
Figure7_RNA-Seq_maturation_signals.R

This pipeline was used for the bulk-RNA-Seq analysis of different culture conditions in vitro vs astrocytes in vivo (related to Figure 7). The first steps of the pipeline are adapted with minor modifications from 'Figure2_RNA-Seq_cortex'

Input files required:

count matrices (in '00_input'): **matrix_FGF_3D.csv**, **matrix_in vitro in vivo_old X6 data.csv**
Output from mapping pipeline crickbabs/BABS-RNASeq; https://github.com/crickbabs/BABS-RNASeq; git commit id: 335ce47db079d6cc2a7f82f4b762620c4f7f27e2; Raw count matrices from culture conditions in vitro experiment and in vivo cortical astrocyte data as reference

In '00_input' folder (provided):
- **group_tab.csv:** *for each sample specification of sample name (from matrix.csv), experimental group*
- **comparisons.csv:** experimental groups to compare in DESeq2 analysis
- **Gene of interest table 21-02-19.csv:** *collection of various gene sets for analyses and plotting (e.g. transcription factors, cell type markers, …)*

The exact reproduction of the analysis also requires:
- **07_Cor vs Str immat mat genes_genes.csv:** *gene sets including the common immature and mature signature (output from 'Figure1_scRNA-Seq_striatum')*

Workflow description:

Part 1 (lines 1 - 165): DESeq analysis as in 'Figure2_RNA-Seq_cortex'
The count matrix for the DESeq analysis was generated from the two input matrices in lines 35-46

Part 2 (lines 166 - 331): Analysis of expression changes of immature/mature astrocyte gene sets in astrocytes in vitro in different culture conditions vs in vivo
Binary/qualitative code for differential expression is added to the results table from Part 1 (-1/0/1 for increased/unchanged/decreased expression). Selected gene sets are manually defined (lines 197 - 219). Statistics for the gene numbers per gene set are generated, and pairwise proportion tests (function pairwise.prop.test()) were used to analyse overlap of all regulated genes with mature/immature gene sets. Heatmaps for the expression of selected gene sets are plotted (lines 322 - 328).

Related figure panels and tables:

Generated from output files indicated (in folder 01_output)

Figures:
7B: 02_heatmap_mature_low_in_bas_2D_TFs_selected.pdf
7C: 02_heatmap_mature_low_in_bas_2D_reg_by_any_signal_selected.pdf, 02_genes_in_modules_stats.csv

Table S9: 02_genes_in_modules.csv

12. FigureS2A_RNA-Seq_cortex_bulk_vs_Str_pseudobulk

Script:
FigureS2A_RNA-Seq_cortex_bulk_vs_Str_pseudobulk.R

This pipeline was used for the pseudo-bulk-RNA-Seq comparison of striatal and cortical astrocyte maturation (related to Figure S2A/B). The first steps of the pipeline are adapted with minor modifications from 'Figure2_RNA-Seq_cortex'

Input files required:

count matrices (in '00_input'):

- **matrix.csv**: *Output from mapping pipeline crickbabs/BABS-RNASeq; https://github.com/crickbabs/BABS-RNASeq; git commit id: 335ce47db079d6cc2a7f82f4b762620c4f7f27e2; Raw count from culture in vivo cortical astrocyte data (see 'Figure2_RNA-Seq_cortex')*

- **01_pseudo_bulk_counts.csv**: *output from 'Figure1_scRNA-Seq_striatum'*

In '00_input' folder (provided):
- **group_tab.csv:** *for each sample specification of sample name (from matrix.csv), experimental group*
- **comparisons.csv:** experimental groups to compare in DESeq2 analysis
- **Gene of interest table 21-02-19.csv:** *collection of various gene sets for analyses and plotting (e.g. transcription factors, cell type markers, …)*

Workflow description:

Part 1 (lines 1 - 173): DESeq analysis as in 'Figure2_RNA-Seq_cortex'
The count matrix for the DESeq analysis was generated from the two input matrices in lines 41 - 80

Part 2 (lines 174-240): Analysis of expression changes of gene expression changes in cortical vs striatal astrocyte maturation
Binary/qualitative code for differential expression is added to the results table from Part 1 (-1/0/1 for increased/unchanged/decreased expression). Selected gene sets are manually defined (lines 198 - 214). Statistics for the gene numbers per gene set are generated and a table with genes in each set.

Part 3 (lines 240-261): Extraction of normalised counts for cell type markers to estimate contamination with other cell types in cortical bulk samples.

Related figure panels and tables:

Generated from output files indicated (in folder 01_output)

Figures:
S2A: 02_reg_genes_cor_vs_str_pseudobulk_stat.csv
S2B: 03_pseudocounts_cor_vs_str_cell type markers.csv

Table S2: 02_reg_genes_cor_vs_str_pseudobulk.csv

13. FigureS6C_RNA-Seq_BMP_vs_published_in_vitro_astrocytes

Script:
FigureS6C_RNA-Seq_BMP_vs_published_in_vitro_astrocytes.R

This pipeline was used for the comparison of astrocyte maturation in vitro and in vivo with different published astrocyte in vitro models (related to Figure S6C). The first steps of the pipeline are adapted with minor modifications from 'Figure2_RNA-Seq_cortex'

Input files required:

count matrices (in '00_input'): **matrix_in vitro Hasel17 Tiwari18.csv**, **matrix_in vitro in vivo_old X6 data.csv**, **matrix_in vitro X6 resequenced.csv**
Output from mapping pipeline crickbabs/BABS-RNASeq; https://github.com/crickbabs/BABS-RNASeq; git commit id: 335ce47db079d6cc2a7f82f4b762620c4f7f27e2; Raw count matrices from in vitro experiments and in vivo cortical astrocyte data as reference (own data) and published in vitro datasets: GEO: GSE96539, EBI Arrayexpress: E-MTAB-5514*) and in vivo cortical astrocyte data as reference*

In '00_input' folder (provided):
- **group_tab.csv:** *for each sample specification of sample name (from matrix.csv), experimental group*
- **comparisons.csv:** experimental groups to compare in DESeq2 analysis
- **Gene of interest table 21-02-19.csv:** *collection of various gene sets for analyses and plotting (e.g. transcription factors, cell type markers, …)*

The exact reproduction of the analysis also requires:
- **07_Cor vs Str immat mat genes_genes.csv:** *gene sets including the common immature and mature signature (output from 'Figure1_scRNA-Seq_striatum')*

Workflow description:

Part 1 (lines 1 - 175): DESeq analysis as in 'Figure2_RNA-Seq_cortex'
The count matrix for the DESeq analysis was generated from the two input matrices in lines 31-57

Part 2 (lines 176 -318): Analysis of expression changes of immature/mature astrocyte gene sets in astrocytes in vitro in different differentiation protocols vs in vivo
Binary/qualitative code for differential expression is added to the results table from Part 1 (-1/0/1 for increased/unchanged/decreased expression). Selected gene sets are manually defined (lines 205-225). Statistics for the gene numbers per gene set are generated. Heatmaps for the expression of selected gene sets are plotted (lines 316-318).

Related figure panels and tables:

Generated from output files indicated (in folder 01_output)

Figures:
S6C: 03_heatmap_mature_sel_BMP_reg.pdf, 02_genes_in_modules_stats.csv

14. FigureS8_re-analysis_published_ChIP_vs_astrocyte_ATAC-Seq

Script:
FigureS8_re-analysis_published_ChIP_vs_astrocyte_ATAC-Seq.R

This pipeline was used for the comparison of published ChIP-Seq datasets to ATAC- and RNA-Seq data from the transcription factor expression in vitro experiments (related to Figure S8).

Input files required (in '00_input'):

From own in vivo ATAC analysis ('Figure3_ATAC-Seq_in_vivo'):
- **01_ATAC_peaks_full_annot.csv**
- **03_ATAC_RNA_merged_detected_with_binary_comparisons.csv**

From own ATAC analysis of transcription factor expression ('Figure6_ATAC-Seq_transcription_factor_expression'):
- **03_Peaks_Lhx2_opening.bed**
- **03_Peaks_mature_opening.bed**
- **03_Peaks_Rorb_opening.bed**

From own RNA analysis of combined transcription factor expression ('Figure6_RNA-Seq_Rorb_Fezf2_co-expression'):
- **02_genes_in_modules.csv**

Common mature/immature gene signature (from 'Figure1_scRNA-Seq_striatum')
- **07_Cor vs Str immat mat genes_genes.csv**

ChIP-Seq peak datasets (mapped with the nf-core chip-seq pipeline (version 1.2.1)) from Lhx2 in olfactory neurons (GSE93570), Fezf2 in neurospheres (GSE46707), and RORg (Rorc) in Th17 cells, as a proxy for Rorb binding (SRP104092).
- **Fezf2_R1_peaks.annotatePeaks.txt**
- **Fezf2_R2_peaks.annotatePeaks.txt**
- **Lhx2_chip_R1_peaks.annotatePeaks.txt**
- **Lhx2_chip_R2_peaks.annotatePeaks.txt**
- **RORgt_R1_peaks.annotatePeaks.txt**
- **RORgt_R2_peaks.annotatePeaks.txt**

Workflow description:

Part 1 (lines 1 - 130): Annotation of overlapping peaks from other datasets to in vivo ATAC peaks
Based on annotations from '01_ATAC_peaks_full_annot.csv'. Additional annotations (overlapping regions from other ATAC/ChIP datasets, specified in lines 21-56) are added using the annotate_set() function (lines 63-106).

Part 2 (lines 131 - 206): Analysis of overlap between transcription factor binding and chromatin opening
Manual definition of peak sets: ATAC peaks overlapping with ChIP peaks in each both ChIP replicates (Lhx2 or Fezf2 or RORg), or overlapping with chromatin opening upon Lhx2/Rorb overexpression, or both. Bed files for selected comparisons are saved (lines 187-199).

Part 3 (lines 207 - 315): Integration with RNA-Seq analysis of astrocyte maturation upon combined Rorb/Fezf2 overexpression
Manual selection of specific gene sets (genes regulated by Rorb+Fezf2 overexpression, mature astrocyte genes regulated vs not regulated) using '02_genes_in_modules.csv' (lines 222-234). Identification of putative direct target genes (regulated by Fezf2 and Rorb and regions with accessible chromatin in astrocytes that bind Fezf2 and/or RORg in reference sets).
'03_ATAC_RNA_merged_detected_with_binary_comparisons.csv' is used to connect putative regulatory open chromatin regions and target genes. Peak features are manually defined in lines 251 - 296. Gene and peak number statistics are generated.

Related figure panels and tables:

Generated from output files indicated (in folder 01_output)

Figures:
S8C: 02_Stat_ATAC_peakset_overlap_with_reference_sets_extended.csv, 02_Stat_ATAC_peakset_overlap_with_reference_sets.csv
S8F: 03_peak_gene_links_stats.csv, 03_reg_gene_set_stats.csv

Table S8: 01_ATAC_peaks_ext_annot.csv, 02_Stat_ATAC_peakset_overlap_with_reference_sets_extended.csv, 02_Stat_ATAC_peakset_overlap_with_reference_sets.csv, 03_peak_gene_links_stats.csv, 03_reg_gene_set_stats.csv
